# New pentadienone oxime ester derivatives: synthesis and anti-inflammatory activity

**DOI:** 10.1080/14756366.2017.1396455

**Published:** 2017-12-04

**Authors:** Qin Li, Juping Zhang, Liu Zeng Chen, Jie Quan Wang, Hai Ping Zhou, Wen Jian Tang, Wei Xue, Xin Hua Liu

**Affiliations:** a State Key Laboratory Breeding Base of Green Pesticide and Agricultural Bioengineering, Guizhou University, Guiyang, P. R. China;; b School of Pharmacy, Anhui Province Key Laboratory of Major Autoimmune Diseases, Anhui Institute of Innovative Drugs, Anhui Medical University, Hefei, P. R. China;; c School of Material Science Chemical Engineering, ChuZhou University, ChuZhou, P. R. China

**Keywords:** Pentadienone, oxime ester, synthesis, anti-inflammatory, activity

## Abstract

To develop novel anti-inflammatory agents, a series of new pentadienone oxime ester compounds were designed and synthesized. The structures were determined by IR, ^1^H NMR, ^13 ^C NMR, and HRMS. All compounds have been screened for their anti-inflammatory activity by evaluating their inhibition against LPS-induced nitric oxide (NO) release in RAW 264.7 cell. Among them, compound **5j** was found to be one of the most potent compounds in inhibiting NO and IL-6 (IC_50_ values were 6.66 µM and 5.07 µM, respectively). Preliminary mechanism studies show that title compound **5j** could significantly suppress expressions of nitric oxide synthase, COX-2, and NO, IL-6 through Toll-like receptor 4/mitogen-activated protein kinases/NF-κB signalling pathway. These data support further studies to assess rational design of more efficient pentadienone oxime ester derivatives with anti-inflammatory activity in the future.

## Introduction

The body immune response divided into innate and acquired immunity. To cope fatal organisms, body must depend on immune response. Immune response is closely related to inflammation^1^. Inflammation substantially contributes to the development and progression of malignancies. Tumour microenvironment, products of inflammatory cells influence almost every aspect of tumourigenesis and tumour progression. Innate immunity do not have the capacity of discriminating between self and foreign pathogens through a family of evolutionarily conserved receptors known like the Toll-like receptors (TLRs)^2^. Recently, much attention has been focused on the role of TLRs in the pathogenesis of many diseases. For example, Toll-like receptor 4 (TLR4) express has been indicated to play an important role in the regulation anti-inflammatory signalling^3^.

Upon stimulation with LPS, TLR4 initiates a series of signalling cascades resulting in activation of NF-κB and mitogen-activated protein kinases (MAPK) to induce the release of pro-inflammatory cytokines^4^. Recent studies found that inhibition of TLR4 could decrease the expressions of IL-6, IL-1β, and TNF-α, which were consistent with inflammatory^5^. So, the TLR4 signal pathway may be a target in the treatment of inflammatory disease. NF-κB, downstream protein of TLR4, is a key activator in the process of inflammation, it turns on transcriptions of inflammatory genes including TNF-α and IL-6. Meanwhile, MAPK (containing JNK, ERK, and P38), another downstream proteins of TLR4, were quite significant in the regulation of inflammation because of mediating the productions of NO, TNF-α, IL-6, IL-β, and other inflammatory mediators^6^.

Curcumin analogues, has been extensively investigated for centuries in a variety of pharmaceutical applications^7–9^ such as anti-inflammatory and^10–12^ inhibitory activity of COX^13^. Due to its relatively low bioavailability, based on its key moiety, a lot of optimization designs were carried out. Among them, many pentadienone derivatives were synthesized. Kok Wai Lam group has developed the synthesis of symmetrical curcumin (pentadienone analogues) and evaluated their effects on a variety of biological activities including anti-inflammatory and immunomodulatory^14–18^. The results suggested that unsymmetrical form (pentadienone analogues) might possess greater biological profile compared to the symmetrical form^19–21^. Motivated by these findings, in order to find potent anti-inflammatory agents, a series of unsymmetrical diarylpentadienones have been designed and synthesized by us. The results revealed that one compound exhibited strong anti-inflammatory activity on decreasing IL-6, but only lightly reduce activation of p65 in nuclei, and that, this compound has strong cytotoxicity^22^. Recently, oxime ester derivatives have attracted considerable attention in medicinal research due to their anti-inflammatory. A large number of investigations on their synthesis and biological activities have been reported^23–25^.

Herein, in continuation to extend our research for finding new, potent anti-inflammatory agents with higher efficacy and better safety, a series of phenyl-penta-1,4-dien-3-one *O*-benzoyl oxime derivatives were synthesized. Their anti-inflammatory activity was also evaluated.

## Experimental section

### Chemistry

The reactions were monitored by thin layer chromatography (TLC) on pre-coated silica GF254 plates. Melting points were determined on a XT4MP apparatus (Taike Corp., Beijing, China), and are uncorrected. The infrared spectra were determined by IR-960 (Shimadzu, Japan). ^1^H and ^13 ^C NMR spectra were recorded on a Brucker AM-500 (500 MHz) spectrometer with CDCl_3_ or DMSO-*d_6_* as the solvent. High-resolution electron impact mass spectra (HR-MS) were recorded under electron impact using a MicroMass GCT CA 055 instrument, USA.

### General procedure for the synthesis of title compound **5a**

(1E,3E,4E)-1–(4-chlorophenyl)-5–(2-(3-methylbenzyloxy)phenyl)penta-1,4-dien-3-one oxime (compound **4a**) (10 mmol) and potassium carbonate (15 mmol) were dissolved in acetone (10 ml) at room temperature. After 10 min, benzoyl chloride (12 mmol) was slowly drip under the ice bath. The mixture was reacted for 5 h at 0–5 °C.

**Scheme 1.**
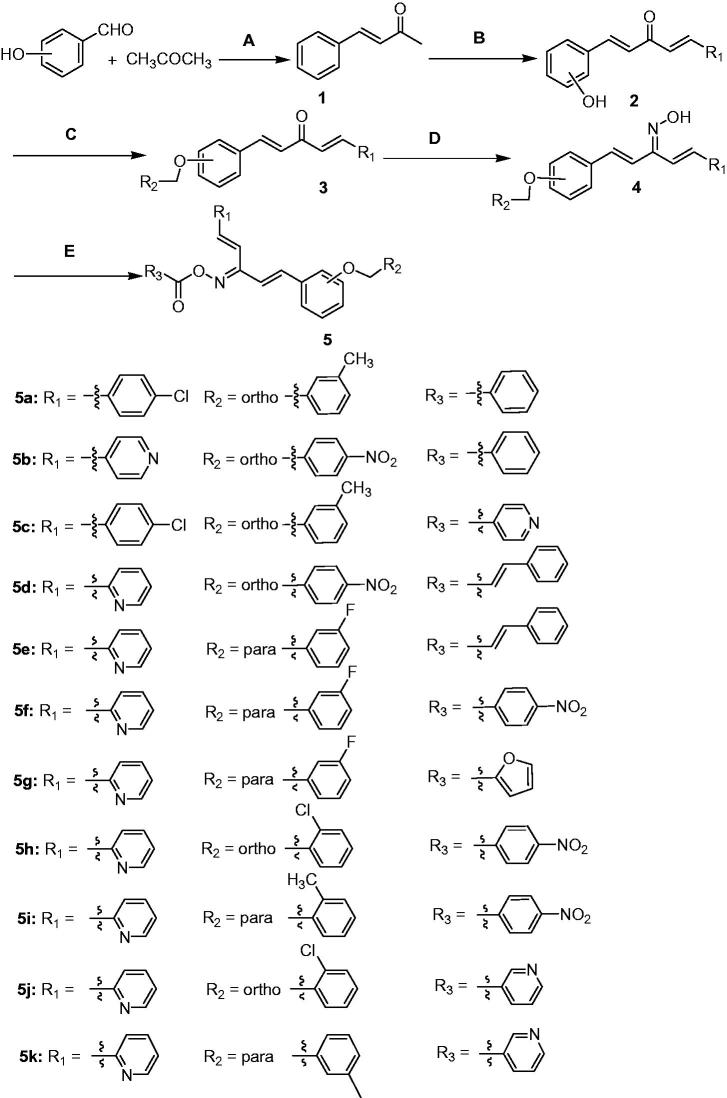
Synthesis of title compounds **5a**–**5k**.

The reaction was monitored by TLC (PE/EA =3/1), a large yellow solid was obtained. The mixture was cooled and collected by filtration, then the crude residue was purified by recrystallization with ethyl acetate to give title compound **5a** (Scheme 1) as yellow solids. According to the same way, compounds **5b**–**5k** were synthesized.

#### 5a

*(1E,3E,4E)-1–(4-chlorophenyl)-5–(2-(3-methylbenzyloxy)phenyl)penta-1,4-dien-3-one O-benzoyl oxime*, Yellow solids, yield, 65.8%, m.p. 1 4 3–145 °C; IR (KBr, cm^−1^) *ν*_max_: 3058, 2851, 1745, 1613, 1593, 1536, 1490, 1452, 1237, 1173, 1053, 1023, 916, 814, 757. ^1^H NMR (500 MHz, DMSO-*d_6_*, ppm) δ 7.99 (dt, *J* = 7.8, 3.8 Hz, 2H, ArH), 7.78 (dd, *J_1_* = 7.7 Hz, *J_2_* = 1.5 Hz, 1H, PyH), 7.73–7.68 (m, 2H, ArH), 7.65 (d, *J* = 9.8 Hz, 1H, Ar-CH=), 7.57 (dd, *J_1_*=16.2 Hz, *J_2_*=10.1 Hz, 2H, ArH), 7.52–7.47 (m, 2H, ArH), 7.44 (d, *J* = 8.5 Hz, 2H, PyH), 7.37 (d, *J* = 12.5 Hz, 1H, Py-CH=), 7.34–7.30 (m, 1H, ArH), 7.23 (d, *J* = 16.2 Hz, 1H, PyH), 7.19–7.13 (m, 3H, ArH), 7.05–6.96 (m, 3H, Ar-C = CH, Py-C = CH, ArH), 5.16 (s, 2H, CH_2_), 2.08 (s, 3H, CH_3_). ^13 ^C NMR (125 MHz, DMSO-*d_6_*, ppm) δ 167.9, 163.4, 162.2, 157.4, 155.4, 138.1, 137.4, 137.2, 136.8, 135.0, 134.3, 131.9, 129.8, 129.5, 129.2, 128.9, 128.2, 124.8, 124.5, 122.1, 121.7, 117.6, 113.8, 70.3, 21.3.

#### 5b

*(1E,3Z,4E)-1–(2-(4-nitrobenzyloxy)phenyl)-5-(pyridin-2-yl)penta-1,4-dien-3-one O-benzoyl oxime,* Light yellow solids, yield, 61.2%, m.p. 1 8 4–86 °C; IR (KBr, cm^−1^) *ν*_max_: 3061, 3005, 2857, 1740, 1598, 1520, 1485, 1452, 1347, 1308, 1239, 1173, 1080, 1060, 1025, 971, 920, 833, 780, 748. ^1^H NMR (500 MHz, DMSO-*d_6_*, ppm) δ 8.84 (d, *J* = 5.2 Hz, 1H, PyH), 8.36 (t, *J* = 7.3 Hz, 1H, ArH), 8.27 (d, *J* = 7.7 Hz, 1H, ArH), 8.12–8.00 (m, 5H, ArH), 7.79 (m, 8H, ArH, PyH), 7.55–7.49 (m, 2H, Ar-CH=, Py-CH=), 7.48–7.42 (m, 1H, PyH), 7.23 (d, *J* = 8.3 Hz 1H, Ar-C = CH), 7.13 (d, *J* = 7.4 Hz, 1H, Py-C = CH), 5.43 (s, 2H, CH_2_).^13 ^C NMR (125 MHz, DMSO-*d_6_*, ppm) δ 163.2, 160.9, 157.1, 150.6, 147.3, 145.5, 145.1, 143.0, 137.5, 134.2, 132.1, 130.1, 129.7, 129.4, 128.7, 128.4, 125.9, 125.5, 124.4, 123.9, 123.1, 121.9, 117.6, 113.6, 69.1. HRMS C_30_H_23_N_3_O_5_, *m/z*: (calcd.), 506.1708 [M + H]^+^ (505.5207).

#### 5c

*(1E,3E,4E)-1–(4-chlorophenyl)-5–(2-(3-methylbenzyloxy)phenyl)penta-1,4-dien-3-one O-nicotinoyl oxime,* Yellow solids, yield, 48.2%, m.p. 1 2 7–129 °C; IR (KBr, cm^−1^) *ν*_max_: 3101, 2837, 1749, 1590, 1489, 1419, 1343, 1236, 1081, 1015, 971, 915, 813, 777, 758. ^1^H NMR (500 MHz, DMSO-*d_6_*, ppm) δ 9.14 (dd, *J_1_* = 2.2 Hz, *J_2_* = 0.8 Hz, 1H, PyH), 8.81 (dd, *J_1_* = 4.8 Hz, *J_2_* = 1.7 Hz, 1H, PyH), 8.30 (d, *J* = 6.4 Hz, 1H, PyH), 7.84–7.81(m, 1H, PyH), 7.73–7.70 (m, 2H, ArH), 7.66–7.59 (m, 2H, ArH), 7.57–7.51 (m, 1H, ArH), 7.47–7.44 (m, 2H, ArH), 7.38 (d, *J* = 10.1 Hz, 1H, ArH), 7.37 (d, *J* = 5.9 Hz,1H, ArH), 7.26–7.22 (m, 1H, ArH), 7.19–7.13 (m, 3H, Ar-C = CH, ArH), 7.02 (dd, *J_1_* = 11.4 Hz, *J_2_* = 9.8 Hz, 2H, Ar-CH=, Ar-C = CH), 6.95 (d, *J* = 5.6 Hz, 1H, Ar-CH=), 5.13 (s, 2H, CH_2_), 2.12 (s, 3H, CH_3_).^13 ^C NMR (125 MHz, DMSO-*d_6_*, ppm) δ 162.6, 157.4, 154.5, 150.6, 138.1, 137.7, 137.5, 137.1, 135.0, 134.3, 132.0, 129.9, 129.4, 128.8, 128.3, 125.1, 124.8, 124.6, 124.4, 121.9, 121.7, 117.5, 113.8, 70.4, 21.3. HRMS C_31_H_25_ClN_2_O_3_, *m/z*: (calcd.), 509.8852 [M + H]^+^ (508.9948).

#### 5d

*(1E,3Z,4E)-1–(2-(4-nitrobenzyloxy)phenyl)-5-(pyridin-2-yl)penta-1,4-dien-3-one O-cinnamoyl oxime,* Light yellow solids, yield, 75.0%, m.p0.1 6 2–164 °C; IR (KBr, cm^−1^) *ν*_max_: 3039, 2853, 1749, 1518, 1487, 1451, 1347, 1301, 1242, 1119, 976, 893, 760. ^1^H NMR (500 MHz, CDCl_3_, ppm) δ 8.65 (d, *J* = 6.1 Hz, 1H, PyH), 8.07 (dd, *J_1_*=13.1 Hz, *J_2_* = 5.2 Hz, 2H, PyH), 7.88 (d, *J* = 16.0 Hz, 1H, PyH), 7.77–7.69 (m, 3H, Py-CH=, ArH), 7.63–7.53 (m, 5H, ArH, Ar-CH=), 7.47–7.33 (m, 7H, ArH), 7.27 (d, *J* = 8.7 Hz, 1H, ArH), 7.09 (d, *J* = 9.6 Hz, 1H, CH = C), 6.93 (d, *J* = 8.7 Hz, 1H, Py-C = CH), 6.61 (d, *J* = 6.1 Hz, 1H, Ar-C = CH), 5.25 (s, 2H, CH_2_). ^13 ^C NMR (125 MHz, CDCl_3_, ppm) δ 164.7, 160.8, 156.4, 154.2, 150.1, 147.6, 146.7, 144.0, 137.9, 136.9, 135.2, 134.3, 131.3, 130.8, 129.1, 128.4, 127.8, 127.4, 125.0, 124.8, 124.0, 123.6, 123.2, 121.9, 117.3, 115.6, 112.5, 69.1. HRMS C_32_H_25_N_3_O_5_, *m/z*: (calcd.), 532.1865 [M + H]^+^ (531.5580).

#### 5e

*(1E,3Z,4E)-1–(4-(3-fluorobenzyloxy)phenyl)-5-(pyridin-2-yl)penta-1,4-dien-3-one O-cinnamoyl oxime*, Light yellow solids, yield, 69.8%, m.p0.1 4 2–144 °C; IR (KBr, cm^−1^) *ν*_max_: 3087, 2951, 1734, 1601, 1508, 1470, 1430, 1378, 1304, 1239, 1175, 1112, 977, 808, 781, 762. ^1^H NMR (500 MHz, CDCl_3_, ppm) δ 8.67 (d, *J* = 25.6 Hz, 1H, PyH), 7.90 (d, *J* = 15.9 Hz, 1H, ArH), 7.70 (t, *J* = 7.5 Hz, 1H, PyH), 7.56 (m, 5H, Ar-CH=, Py-CH=, ArH), 7.45 (m, 7H, PyH, ArH), 7.31 (m, 4H, ArH), 7.23 (d, *J* = 7.9 Hz, 1H, Ar-C = CH), 6.98 (m, 2H, Py-C = CH, ArH), 6.65 (d, *J* = 16.0 Hz, 1H, Ar-C = CH), 5.07 (s, 2H, CH_2_). ^13 ^C NMR (125 MHz, CDCl_3_, ppm) δ 164.8, 160.3, 159.9, 154.2, 149.9, 146.6, 140.0, 138.7, 137.3, 136.9, 134.7, 134.4, 130.8, 130.1, 129.5, 129.1, 128.8, 128.4, 127.5, 125.5, 124.4, 123.5, 115.7, 115.3, 114.9, 69.3. HRMS C_32_H_25_FN_2_O_3_, *m/z*: (calcd.), 527.6256 [M + Na]^+^ (504.5509).

#### 5f

*(1E,3Z,4E)-1–(4-(3-fluorobenzyloxy)phenyl)-5-(pyridin-2-yl)penta-1,4-dien-3-one O-4-fluorobenzoyl oxime,* Yellow solids, yield, 69.2%, m.p0.1 5 3–155 °C; IR (KBr, cm^−1^) *ν*_max_: 3089, 3003, 2851, 1731, 1586, 1513, 1474, 1426, 1272, 1171, 1069, 1011, 945, 907, 843, 746. ^1^H NMR (500 MHz, DMSO-*d_6_*, ppm) δ 8.73 (d, *J* = 7.2 Hz,1H, PyH), 8.24–8.11 (m, 3H, ArH), 8.07 (d, *J* = 8.5 Hz, 1H, PyH), 7.77 (m, 3H, ArH, PyH), 7.60 (d, *J* = 8.4 Hz, 1H, PyH), 7.54–7.45 (m, 3H, ArH, Py-CH=), 7.40 (m, 6H, ArH, Ar-CH=), 7.15–6.99 (m, 2H, Ar-C = CH, Py-C = CH), 5.14 (s, 2H, CH_2_).^13 ^C NMR (125 MHz, DMSO-*d_6_*, ppm) δ 162.5, 160.7, 160.3, 141.7, 139.9, 133.7, 133.1, 133.0, 131.0, 130.5, 128.7, 128.4, 127.9, 126.8, 125.6, 125.5, 125.4, 125.3, 125.2, 125.1, 125.0, 116.9, 116.7, 115.8, 114.2, 68.9. HRMS C_30_H_22_F_2_N_2_O_3_, *m/z*: (calcd.), 497.3353 [M + H]^+^(496.5040).

#### 5g

*(1E,3Z,4E)-1–(4-(3-fluorobenzyloxy)phenyl)-5-(pyridin-2-yl)penta-1,4-dien-3-one O-furan-2-carbonyl oxime,* Colourless solids, yield, 76.0%, m.p0.1 4 4–145 °C; IR (KBr, cm^−1^) *ν*_max_: 3128, 3039, 2853, 1752, 1620, 1574, 1487, 1469, 1349, 1285, 1235, 1171, 1191, 969, 901, 883, 857, 799, 768, 742. ^1^H NMR (500 MHz, DMSO-*d_6,_* ppm) δ 8.63 (dd, *J_1_*=16.2 Hz, *J_2_* = 4.5 Hz, 1H, PyH), 8.05 (d, *J* = 13.1 Hz, 1H, TeH), 7.82 (dd, *J_1_*=14.7 Hz, *J_2_*=7.3 Hz, 2H, ArH), 7.69–7.57 (m, 2H, TeH, ArH), 7.57–7.32 (m, 8H, ArH, TeH, PyH), 7.29–7.21 (m, 2H, ArH, Py-CH=), 7.12 (d, *J* = 7.5 Hz, 1H, Ar-CH=), 7.06 (d, *J* = 7.5 Hz, 1H, Py-C = CH), 6.70 (d, *J* = 8.7 Hz, 1H, Ar-C = CH), 5.23 (s, 2H, CH_2_). ^13 ^C NMR (125 MHz, DMSO-*d_6_*, ppm) δ 161.5, 157.0, 155.7, 153.7, 150.4, 149.1, 142.5, 138.1, 137.7, 136.3, 134.5, 133.1, 132.1, 130.5, 130.4, 130.0, 128.7, 127.7, 124.5, 124.4, 124.3, 124.2, 122.1, 120.1, 117.1, 114.0, 113.1, 68.1. HRMS C_28_H_21_FN_2_O_4_, *m/z*: (calcd.), 491.2989 [M + Na]^+^ (468.4757).

#### 5h

*(1E,3Z,4E)-1–(2-(2-chlorobenzyloxy)phenyl)-5-(pyridin-2-yl)penta-1,4-dien-3-one O-4-fluorobenzoyl oxime*, Colourless solids, yield, 59.1%, m.p0.1 6 0–161 °C; IR (KBr, cm^−1^) *ν*_max_: 3058, 2873, 1747, 1601, 1485, 1454, 1347, 1302, 1241, 1157, 1126, 1062, 972, 919, 855, 774, 746. ^1^H NMR (500 MHz, DMSO-*d_6_*, ppm) δ 8.61 (d, *J* = 4.0 Hz, 1H, PyH), 8.13–8.02 (m, 2H, ArH), 7.88–7.78 (m, 2H, ArH), 7.62 (dd, *J_1_*=15.7 Hz, *J_2_*=12.3 Hz, 2H, PyH), 7.56–7.48 (m, 3H, ArH), 7.42–7.30 (m, 6H, Py-CH=, ArH), 7.25–7.19 (m, 2H, Ar-CH=, ArH), 7.11–7.02 (m, 2H, Ar-C = CH, Py-C = CH), 5.21 (s, 2H, CH_2_). ^13 ^C NMR (125 MHz, DMSO-*d_6_*, ppm) δ 162.5, 161.8, 157.0, 153.7, 150.4, 138.2, 137.7, 136.2, 134.5, 133.1, 132.9, 132.8, 132.1, 130.3, 129.9, 128.9, 127.7, 125.4, 124.6, 124.5, 124.4, 124.3, 122.0, 117.2, 116.8, 116.6, 114.0, 68.1. HRMS C_30_H_22_ClFN_2_O_3_, *m/z*: (calcd.), 514.1406 [M + H]^+^(512.9586).

#### 5i

*(1E,3Z,4E)-1–(4-(2-methylbenzyloxy)phenyl)-5-(pyridin-2-yl)penta-1,4-dien-3-one O-4-fluorobenzoyl oxime,* Light yellow solids, yield, 58.0%, m.p0.9 7 ∼ 98 °C; IR (KBr, cm^−1^) *ν*_max_: 3031, 2871, 1757, 1602, 1506, 1466, 1430, 1341, 1246, 1176, 1066, 1003, 959, 916, 847, 815, 750. ^1^H NMR (500 MHz, DMSO-*d_6_*, ppm) δ 8.62 (d, *J* = 4.4 Hz, 1H, PyH), 8.15 (dt, *J* = 20.3 Hz, 2H, ArH), 7.83 (t, *J* = 7.6 Hz, 1H, PyH), 7.74 (dd, *J_1_* = 14.4 Hz, *J_2_* = 8.3 Hz, 3H, ArH), 7.57 (d, *J* = 15.7 Hz, 1H, Py-CH=), 7.47–7.32 (m, 5H, PyH, ArH), 7.29–7.16 (m, 4H, ArH), 7.14–6.97 (m, 3H, Ar-C = CH, Py-C = CH, Ar-CH=), 5.08 (s, 2H, CH_2_), 2.26 (s, 3H, CH_3_).^13 ^C NMR (125 MHz, DMSO-*d_6_*, ppm) δ 162.7, 161.3, 160.5, 154.0, 150.3, 141.3, 138.2, 137.7, 137.7, 137.2, 133.0, 132.9, 130.4, 130.4, 129.1, 128.9, 128.8, 128.5, 125.4, 124.4, 124.3, 116.9, 116.7, 115.8, 114.3, 69.9, 21.5. HRMS C_31_H_25_FN_2_O_3_, *m/z*: (calcd.), 493.1921 [M + H]^+^(492.5402).

#### 5j

*(1E,3Z,4E)-1–(2-(2-chlorobenzyloxy)phenyl)-5-(pyridin-2-yl)penta-1,4-dien-3-one O-nicotinoyl oxime*, Light yellow solids, yield, 71.4%, m.p0.1 5 4–156 °C; IR (KBr, cm^−1^) *ν*_max_: 3035, 2883, 1756, 1586, 1487, 1435, 1349, 1244, 1190, 1080, 1018, 971, 916, 830, 774, 749. ^1^H NMR (500 MHz, DMSO-*d_6_*, ppm) δ 9.15 (d, *J* = 4.3 Hz, 1H, PyH), 8.83 (dd, *J_1_* = 4.8 Hz, *J_2_* = 1.6 Hz, 1H, PyH), 8.63 (d, *J* = 11.6 Hz, 1H, PyH), 8.34 (d, *J* = 6.4 Hz, 1H, PyH), 7.95–7.78 (m, 2H, ArH), 7.70–7.48 (m, 6H, PyH, ArH), 7.43–7.31 (m, 4H, ArH, Py-CH=), 7.26–7.18 (m, 2H, PyH, Ar-CH=), 7.10–7.02 (m, 2H, Ar-C = CH, Py-C = CH), 5.26 (s, 2H, CH_2_). ^13 ^C NMR (125 MHz, DMSO-*d_6_*, ppm) δ 162.5, 162.2, 157.0, 154.5, 153.7, 150.7, 150.4, 138.4, 137.7, 137.6, 136.4, 134.5, 133.1, 132.2, 130.4, 130.3, 129.9, 128.8, 127.7, 125.1, 124.6, 124.5, 124.4, 124.3, 124.2, 122.0, 117.2, 114.0, 68.1. HRMS C_29_H_22_ClN_3_O_3_, *m/z*: (calcd.), 496.1421 [M + H]^+^(495.9562).

#### 5k

*(1E,3Z,4E)-1–(4-(3-methylbenzyloxy)phenyl)-5-(pyridin-2-yl)penta-1,4-dien-3-one O-nicotinoyl oxime,* Brown solids, yield, 59.4%, m.p0.1 1 6–118 °C; IR (KBr, cm^−1^) *ν*_max_: 3051, 3001, 2859, 1754, 1601, 1507, 1465, 1422, 1306, 1260, 1173, 1076, 1017, 970, 918, 826, 774. ^1^H NMR (500 MHz, DMSO-*d_6_*, ppm) δ 9.29–9.14 (d, *J* = 5.2 Hz, 1H, PyH), 8.84 (dt, *J* = 4.5 Hz, 1H, PyH), 8.64 (dd, *J_1_*=12.3 Hz, *J_2_*=4.7 Hz, 1H, PyH), 8.50–8.37 (m, 1H, PyH), 7.83 (td, *J* = 7.6 Hz, 1H, ArH), 7.76–7.67 (m, 3H, ArH), 7.63–7.54 (m, 2H, PyH), 7.43–7.31 (m, 4H, Py-CH=, ArH), 7.27–7.17 (m, 3H, ArH), 7.11 (d, *J* = 7.2 Hz, 1H, Ar-CH=), 7.03 (m, 2H, Py-C = CH, Ar-CH=), 5.08 (s, 2H, CH_2_), 2.26 (s, 3H, CH_3_).^13 ^C NMR (125 MHz, DMSO-*d_6_*, ppm) δ 162.6, 161.7, 160.5, 154.5, 153.9, 150.7, 150.4, 141.4, 138.2, 137.9, 137.7, 137.6, 137.2, 130.5, 129.1, 128.9, 128.8, 128.5, 125.4, 125.3, 124.7, 124.4, 124.3, 124.2, 115.7, 114.4, 69.9, 21.5. HRMS C_30_H_25_N_3_O_3_, *m/z*: (calcd.), 476.1966 [M + H]^+^(475.5378).

### Cell culture

Murine monocyte-macrophage RAW264.7 cells maintained in Dulbecco’s modified Eagle’s medium (DMEM, Hyclone, Miami, FL) supplemented with 10% foetal bovine serum (Beyotime, Shanghai, China), 100 units/mL penicillin, and 100 mg/mL streptomycin and incubated at 37 °C in a humidified atmosphere containing 5% CO_2_.

### Cell viability assay

Cell cytotoxicity was evaluated by MTT assay as previous reported. The medium was changed before the assay. MTT dissolved in phosphate buffered saline and was added to the culture medium to reach a final concentration of 0.5 mg/ml. After incubation at 37 °C for 4 h, the culture media containing MTT were removed, and then DMSO was added into each well and the absorbance at 570 nm was measured by a microplate reader (MQX200, Bio-Tek, Winooski, VT).

### Assay for NO production

NO production was quantified by nitrite accumulation in the culture medium using the Griess reaction. Briefly, RAW264.7 cells were pretreated with compounds for 1 h, and then stimulated with or without LPS (1 µg/ml) for 24 h. The isolated supernatants were mixed with an equal volume of Griess reagent (Beyotime Biotechnology, China). NaNO_2_ was used to generate a standard curve, and nitrite production was determined by measuring the optical density at 540 nm by a microplate reader (MQX200, Bio-Tek).

### Measurement of cytokine production

Cytokine production was measured by ELISA. In brief, RAW264.7 macrophage cells (7 × 10^4^ cells/well) were plated in 24-well plates. After incubation for 24 h, cells were starved by being cultured in serum-free medium for another 2.5 h to eliminate FBS influence. The cells were then treated with compounds for 1 h before exposure to 1 µg/mL LPS for 24 h. The culture medium was used to assay the cytokine production with mouse ELISA kit (IL-6: R&D SYSTEMS, DY406–05) according to manufacturer’s instructions.

### Western blotting

Western blotting assay was performed as described previously. Briefly, RAW264.7 cells (2 × 10^6^ cells/well) were cultured in 6 cm dishes for 24 h, cells were starved by being cultured in serum-free medium for another 2.5 h to eliminate influence of FBS. The cells were then treated with or without compounds for 1 h before exposure to LPS for the indicated times. The cells were lysed in 240 µL RIPA cell lysis buffer (Beyotime, China), and incubated on ice for 30 min. Approximate 40 mg of proteins were run on 10% SDSPAGE and then transferred to PVDF membrane (GE Healthcare, UK). The blotted membrane was incubated with specific primary antibody for overnight at 4 °C and further incubated for 1 h with HRP-conjugated secondary antibody.

### Immunofluorescence analysis

RAW264.7 cells were grown on glass coverslip in six well plates, fixed with 4% paraformaldehyde (w/v) for 20 m at room temperature and blocked for 1 h with 5% BSA in TBS containing 0.1% Triton X-100. Then, the cells were incubated with a primary antibody, followed by Alexa Fluor 488-labeledgoatanti-rabbit IgG. After a wash step, they were stained with DAPI and the images were acquired.

### Statistical analysis

Results are expressed as the mean values SD and were analysed statistically with analysis of variance, and differences between groups were assessed with the Tukey’s method. A value of *p* < .05 was considered to be statistically significant. IR, ^1^H NMR, ^13^C NMR and HRMS (Compounds **5a**∼**5k**) contains the supplementary data.

## Results and discussion

### Chemistry

Claisen Schmidt condensation substituted benzaldehyde and acetone using catalyst sodium hydroxide (5%), proved to be an efficient method for the synthesis of α, β unsaturated ketone (compound **1**). According to the same method, 1,5-penta-1,4-dien-3-ones were synthesized (compound **2**)^26^. The preparation of oxime was the key step for the synthesis of title compounds. The compounds **3** were converted into their corresponding oximes by hydroxylamine^27^. Spectroscopic studies of the oximes and the oxime esters in solution confirmed the presence of anti-configuration. In order to optimize the reaction conditions, the synthesis were carried out in presence of different bases. It was found that yields up to 80% when the reaction was refluxed with ethanol for 20 h catalysed by pyridine (Scheme 1).

#### Reagents and conditions

(A) NaOH, EtOH; (B) aldehyde, NaOH, EtOH; (C) benzyl chloride, K_2_CO_3_, KI, acetone; (D) NH_2_OH**^.^**HCl, EtOH, pyridine; (E) acyl chloride, K_2_CO_3_, acetone.

### Initial evaluation release of NO and IL-6 LPS-induced

**Figure 1.**
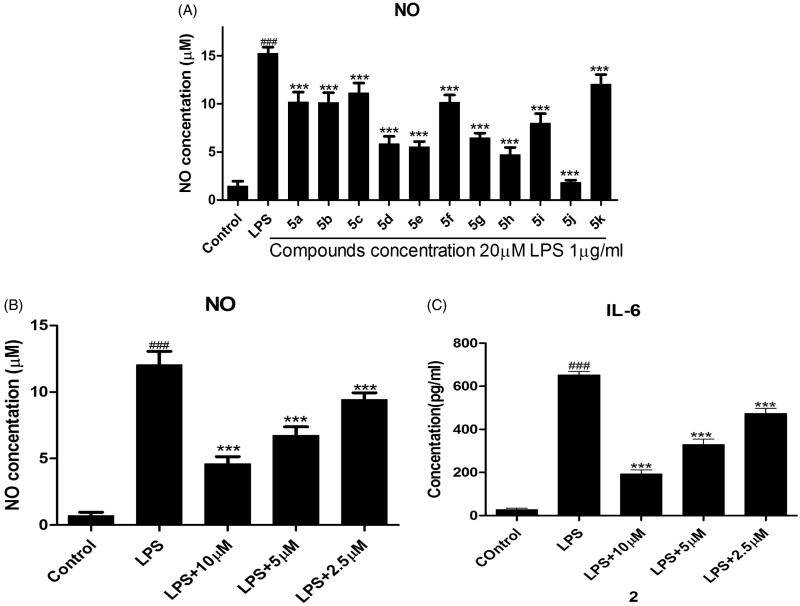
Initial evaluation release of NO and IL-6.

The pro-inflammatory mediators NO and IL-6 play an important roles in the course of inflammation-related diseases^28^. It has been proven that NO inhibitors may offer potential opportunity to identify new therapeutic method for the inflammatory diseases. Hence, in the preliminary screening studies of anti-inflammatory activity, the enzyme-linked immunosorbent assay (ELISA) and nitrate assay was used to screen the inhibition of all compounds (**5a–5k**) toward LPS-induced NO and IL-6 release in RAW264.7 mouse macrophages. The ability of the tested compounds to reduce proinflammatory cytokines NO was summarized in Figure 1(A).

Among them, compounds **5d**, **5e**, **5 h**, and **5j** exhibited similar or better NO inhibitory activity than other compounds. Particularly, compound **5j** exhibited the most potent inhibitory activity and its NO inhibition rate was 92.85% at the concentration of 20 µM. When the concentration of compound **5j** is 2.5 µM to 10 µM, the IC_50_ values of NO and IL-6 were observed for 6.66 µM and 5.07 µM, respectively (Figure 1(B,C)). The preliminary structure activity relationships showed that substituent **R^3^** possessed large effect on the activity, pyridine ring is better than that of benzene (compound **5j**). Further, substituent **R^2^** of benzene showed great influence against inhibit activity (compounds **5d**, **5e**, **5j**). These results are very useful for our next study.

RAW264.7 cells were precultured for 24 h, then the cells were treated with the indicated concentrations of compounds for 1 h, and then exposed to 1 µg/mL LPS for 24 h. The levels of NO and IL-6 in the culture medium were measured by nitrate assay and ELISA. Cells were treated with 20 µM compounds Figure 1(A). Cells were pretreated with different concentrations of compound **5j**Figure 1(B and C). ###*p* < .001 compared with unstimulated cells, ***p < .001, *p < .05 compared with LPS-stimulated cells; data were obtained by at least three independent experiments, and each was performed in duplicate.

### Preliminary mechanism studies

**Figure 2.**
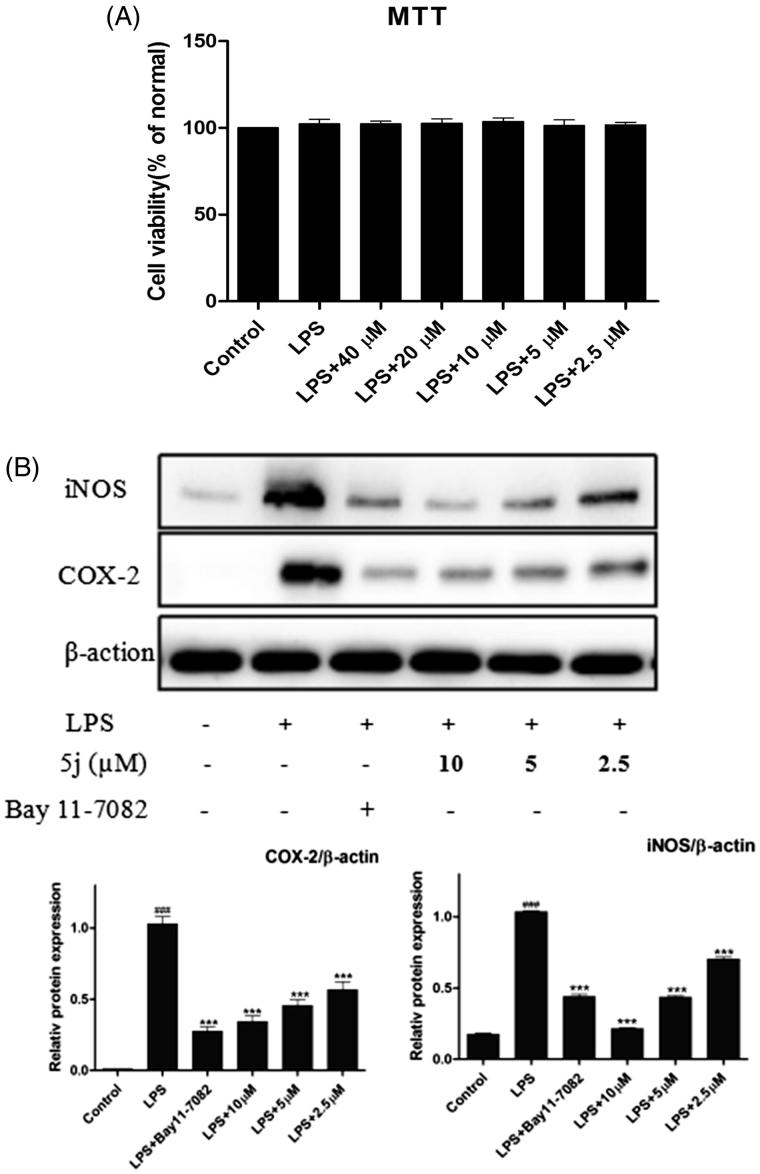
Compound **5j** inhibited expression of iNOS and COX-2.

The pro-inflammatory mediators NO and IL-6 play an important role in inflammation-related diseases, also related to modulation expressions of nitric oxide synthase (iNOS) and COX-2^29^. Persistent-enhanced expressions of iNOS and COX-2 lead to an increased production of pro-inflammatory mediators, which eventually results in the progression of inflammatory responses. Thus, the inhibitory effects of compound **5j** on expressions of iNOS and COX-2 were analysed by Western blot. The cell viability experiment was performed at 4 0 ∼ 2.5 µM and there was no significantly cytotoxic of compound **5j** up to 40 µM. As shown in Figure 2(A), title compound **5j** was also concentration-dependently reduced expressions of iNOS and COX-2 (Figure 2(B)). This results preliminary demonstrated that title compound prevented inflammatory response LPS-induced in RAW264.7 cells.

Treatment with compound **5j**, RAW 264.7 cells were stimulated with LPS (1 µg/mL) for 24 h. Cell viability was evaluated using the MTT assay. Treated with compound (2.5–40 µM), RAW 264.7 cells were stimulated with LPS (1 µg/mL) for 24 h. iNOS and COX-2 expressions were detected by western blot analysis. β-Actin was used as loading control; ###*p* < .001 compared with unstimulated cells, ***p < .001 compared with LPS-stimulated cells; data were obtained by at least three independent experiments, and each was performed in duplicate.

**Figure 3.**
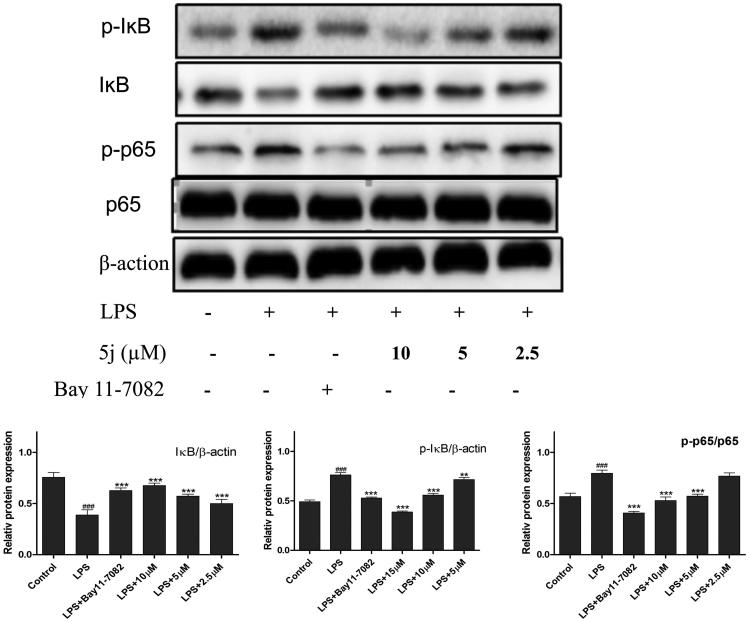
Compound **5j** suppressed activation of NF-κB.

**Figure 4.**
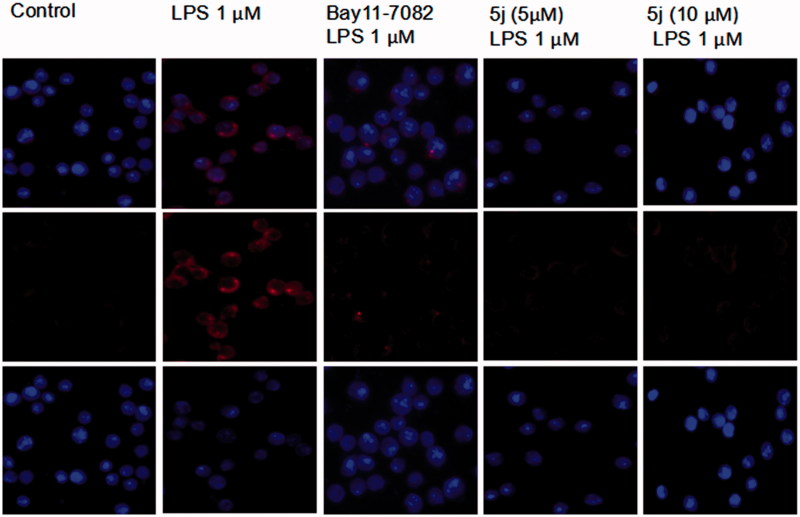
Compound **5j** inhibitory effect on nuclear translocation of p65.

NF-κB is one of the principal factors for the production proinflammatory cytokines^5^. The transcriptional factor NF-κB regulators, is well-known for playing a pivotal role in the initiation and amplification of the inflammatory process^30^. When LPS-stimulated cells NF-κB activation, IκB proteins phosphorylation, and degradation frees NF-κB p65 subunit from sequestration, allowing it to translocate to the nucleus, bind to target promoters, and turn on transcriptions of inflammatory genes including TNF-*α* and IL-6. Herein, the effects of compound **5j** on phosphorylation and degradation of IκB (Figure 3) were screened. Meanwhile it effects on NF-κB p65 translocation from cytoplasm to nuclei were analysed by Western blot and immunofluorescence analysis. The results (Figure 4) showed that NF-κB subunit p65 was almost exclusively observed in the cytoplasm in the un-stimulated cells. After stimulation with LPS (1 µg/mL) for 3 h, most cytoplasmic p65 was translocated into the nucleus. Nuclear localization of p65 was significantly reduced by Bay-117082 (20 µM). Similar result was also found with compound **5j** at all concentrations examined. Consistent with these findings, the levels of p65 were significantly increased in nuclear after LPS-induction for 3 h, which were prevented compound **5j** at all concentrations examined.

Treatment with compound **5j** (2.5–10 µM), RAW 264.7 cells were stimulated with LPS (1 µg/mL) for 30 min. p-IKB IKB, p-p65, p65 and β-actin were detected with their antibodies, respectively. The induction fold of the phosphorylated kinase was calculated as the intensity of the treatment relative to that of control normalized to a-tubulin by densitometry. Bay 11–7082 (20 µM) is the NF-ĸB inhibitors. ###*p* < .001 compared with unstimulated cells, ***p < .001, **p < .01, *p < .05 compared with LPS-stimulated cells; the blots shown are the examples of three separate experiments.

Compound **5j** exhibited potent inhibitory effect on nuclear translocation of p65 in LPS-stimulated RAW264.7 cells. Cells were treated with compound **5j** for 1 h, followed by stimulation with LPS for 3 h. The cellular localization of p65 was determined by immunofluorescence.

**Figure 5.**
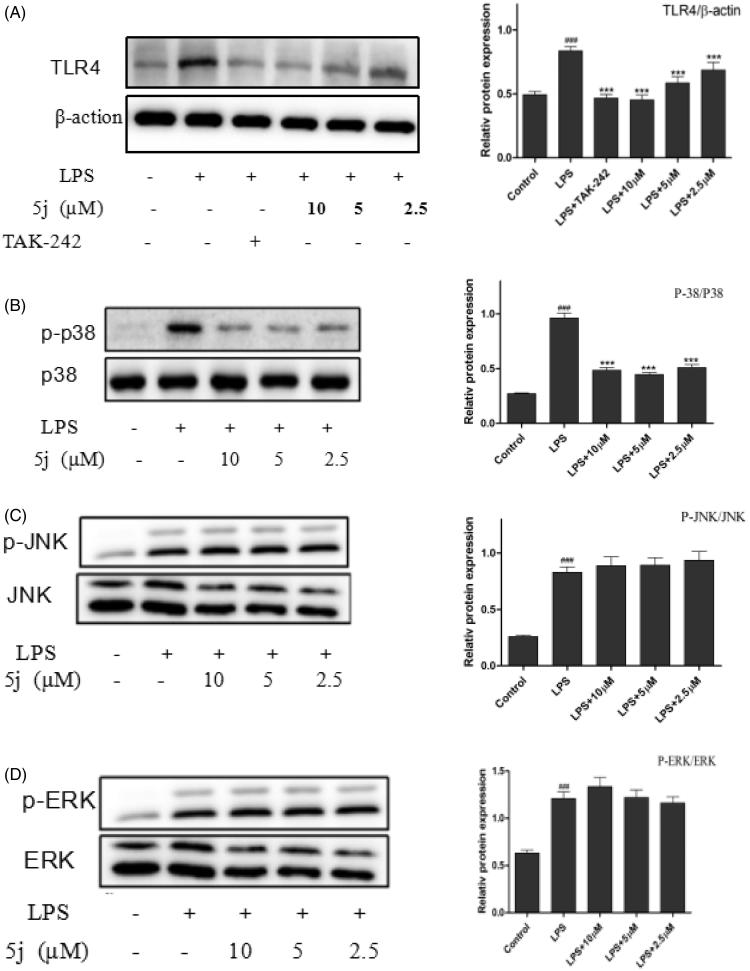
Compound **5j** inhibited expressions of TLR4, P-P38/P38, P-ERK/ERK, P-JNK/JNK.

Currently, 11 toll-like receptor (TLR) family members have been identified in humans and 13 of which are found in mice. TLR4 is an important Toll-like receptor in TLRs family, can be activated by LPS and induce proinflammatory cytokines to resist invasion of pathogenic microorganism, but over inflammation can cause tissue injury. Many genes negatively regulate TLR4 signalling pathway^31^. In addition, TLR4 is traditionally accepted as the primary LPS receptor and has been reported as critical for the inflammatory response to LPS. Upon stimulation with LPS, TLR4 initiates series of signalling cascades that result in the activation of NF-κB and MAPK to induce the release of pro-inflammatory cytokines^10^. MAPK were quite significant in the regulation of inflammation because of their crucial roles in the mediation of the production of NO, TNF-α, IL-6, IL-β, and other inflammatory mediators in activated RAW264.7. Recent studies found that inhibition of TLR4 could decrease the expressions of IL-6, NO, and TNF-α, which was consistent with the improvement of inflammatory. So, we investigated whether compound **5j** inhibited expression of Tak1 by Western blot. The results confirmed that TLR4 expression was strongly up-regulated in LPS-induced RAW264.7 cells (Figure 5(A)). LPS could cause a significant phosphorylation of MAPK (p38, JNK, and ERK) after stimulated for 30 min (Figure 5(B–D)). Compound **5j** diminished LPS-induced phosphorylation of p-p38 (Figure 5(B)), but had little effect on phosphorylation of JNK or ERK (Figure 5(C,D)). These results strongly suggested that inhibition of TLR4 and p-p38 phosphorylation by compound **5j** might be responsible for achieving LPS-induced inflammatory response. Furthermore, compound **5j** also concentration-dependently (2.5 µM, 5 µM, and 10 µM) inhibited LPS-induced ERK phosphorylation in RAW264.7 cells (Figure 5(D)). These results suggest that the anti-inflammatory activity of compound **5j** may be associated with its negative effects on TLR4/p38/NF-κB activation.

RAW264.7 cells were treated with compound **5j** (0.5–2 µM) and LPS (1 µg/mL) for 30 min. The levels of TLR4, P-P38/P38, P-ERK/ERK, P-JNK/JNK and β-actin proteins, and their phosphorylated forms were analysed using Western blotting. TAK-242 (200 nM) is the TLR4 inhibitors. The results were shown as means ± standard deviation (SD) (*n* = 3) of at least three independent experiments. ###*p* < .001 compared with the control group; *p < .05, **p < .01, ***p < .001 compare with LPS-stimulated.

## Conclusions

In summary, based on finding new pentadienone oxime esters with anti-inflammatory activity, 12 derivatives were synthesized. The results of initial evaluation showed that some compounds exhibited good NO and IL-6 inhibitory activity. Among them, (1E,3Z,4E)-1–(2-(2-chlorobenzyloxy)phenyl)-5-(pyridin-2-yl) penta-1,4-dien-3-one O-nicotinoyl oxime (compound **5j)** showed potent anti-inflammatory activity by inhibition the expressions of iNOS, COX-2 and NO, IL-6. The preliminary mechanism of anti-inflammatory action indicated that title compound **5j** could significantly suppress expressions of iNOS, COX-2, and IL-6 production through NF-κB/MAPK signalling pathway. Compared with our previous diarylpentadienone moiety, this oxime ester compound has lower toxicity and better activity.

## Supplementary Material

IENZ_1396455_Supplementary_Material.pdf
