# Supplementary material for: New pentadienone oxime ester derivatives: synthesis and anti-inflammatory activity
Source: J Enzyme Inhib Med Chem. 2017 Dec 4;33(1):130–8. doi: 10.1080/14756366.2017.1396455 (PMC6010105; doi:10.1080/14756366.2017.1396455)
Supplement: IENZ_1396455_Supplementary_Material.pdf [file IENZ_A_1396455_SM8638.pdf]

5a

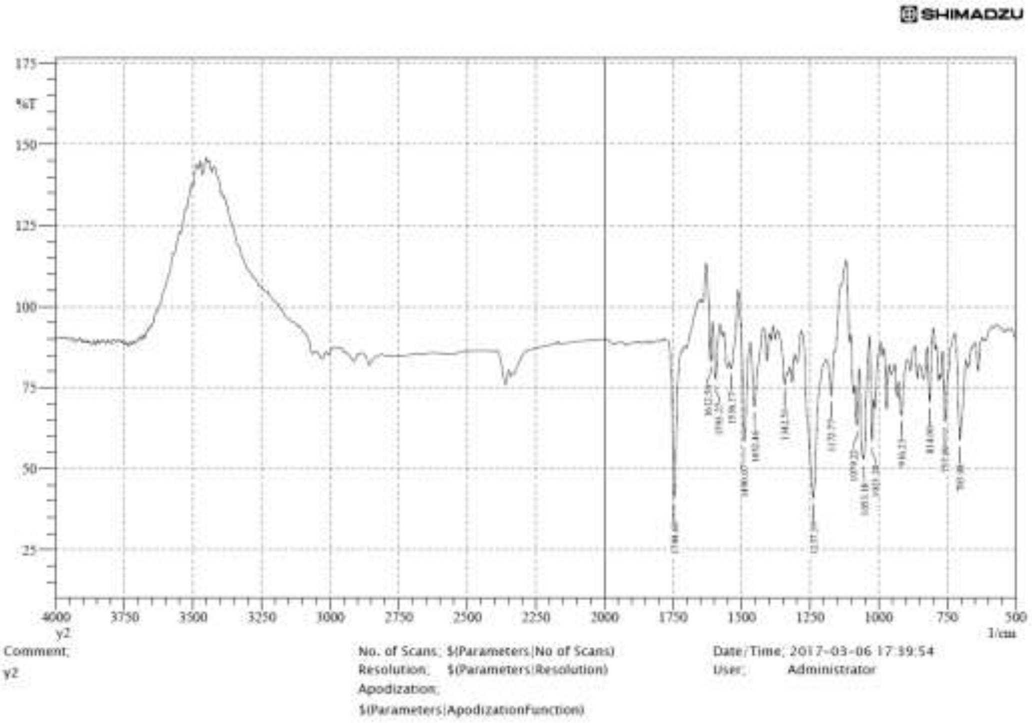
SHIMADZU
%T
1/cm
Comment;
y2
No. of Scans; $(Parameters|No of Scans)
Resolution; $(Parameters|Resolution)
Apodization;
$(Parameters|ApodizationFunction)
Date/Time; 2017-03-06 17:39:54
User; Administrator

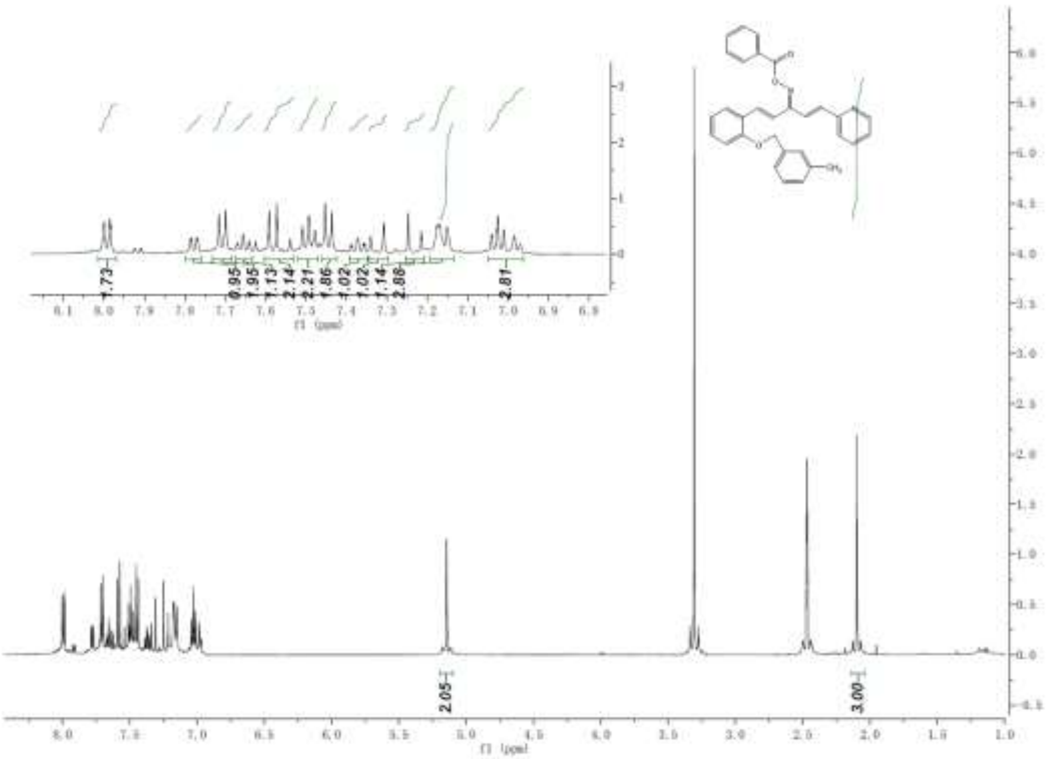
1.73
0.95
1.95
1.13
2.14
2.21
1.86
1.02
1.02
1.14
2.88
2.81
f1 (ppm)
2.05
3.00
f1 (ppm)

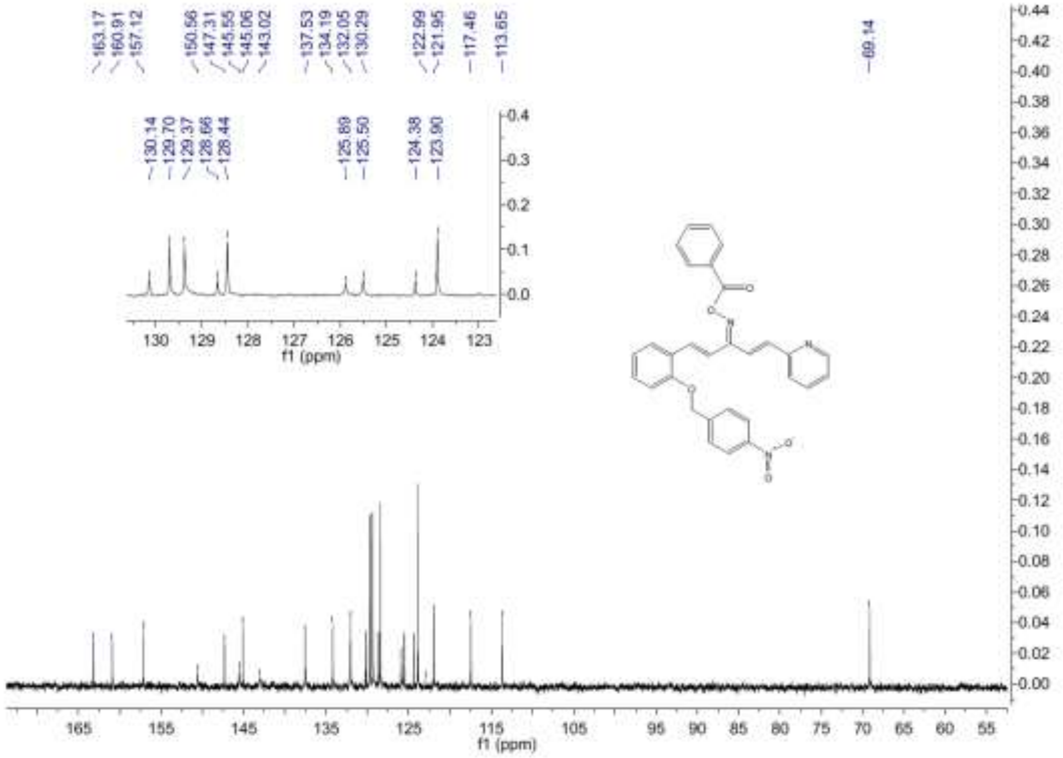
163.17
160.91
157.12
150.56
147.31
145.55
145.06
143.02
137.53
134.19
132.05
130.29
122.99
121.95
117.46
113.65
69.14
130.14
129.70
129.37
128.66
128.44
125.89
125.50
124.38
123.90
f1 (ppm)
f1 (ppm)

5b:

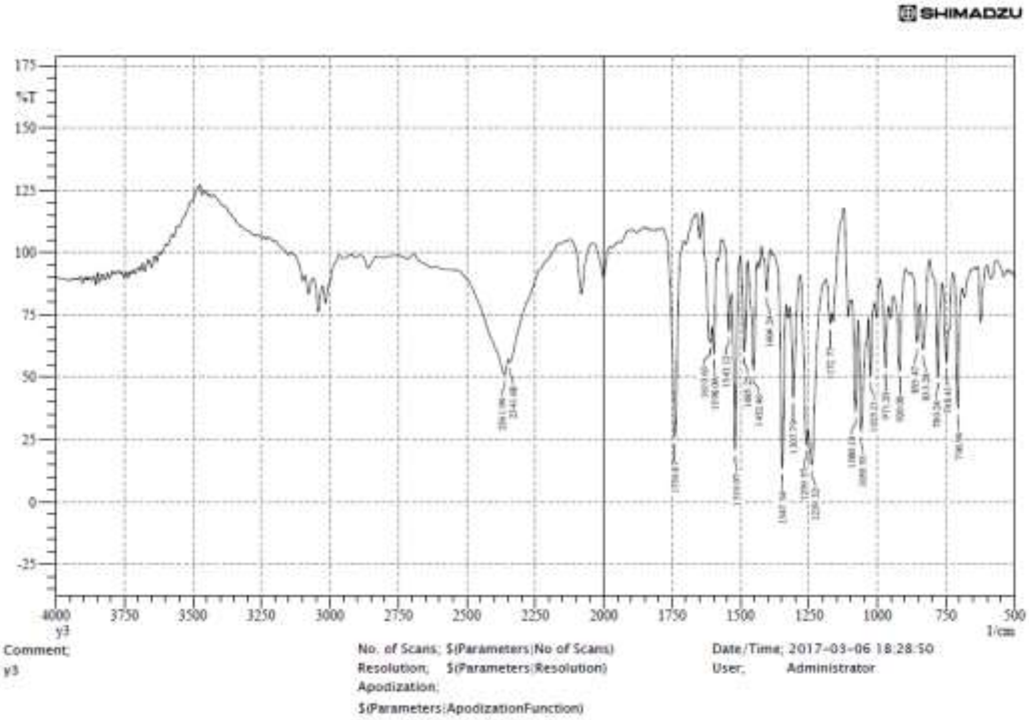
SHIMADZU
%T
1/cm
y3
Comment;
y3
No. of Scans; $(Parameters|No of Scans)
Resolution; $(Parameters|Resolution)
Apodization;
$(Parameters|ApodizationFunction)
Date/Time; 2017-03-06 18:28:50
User; Administrator

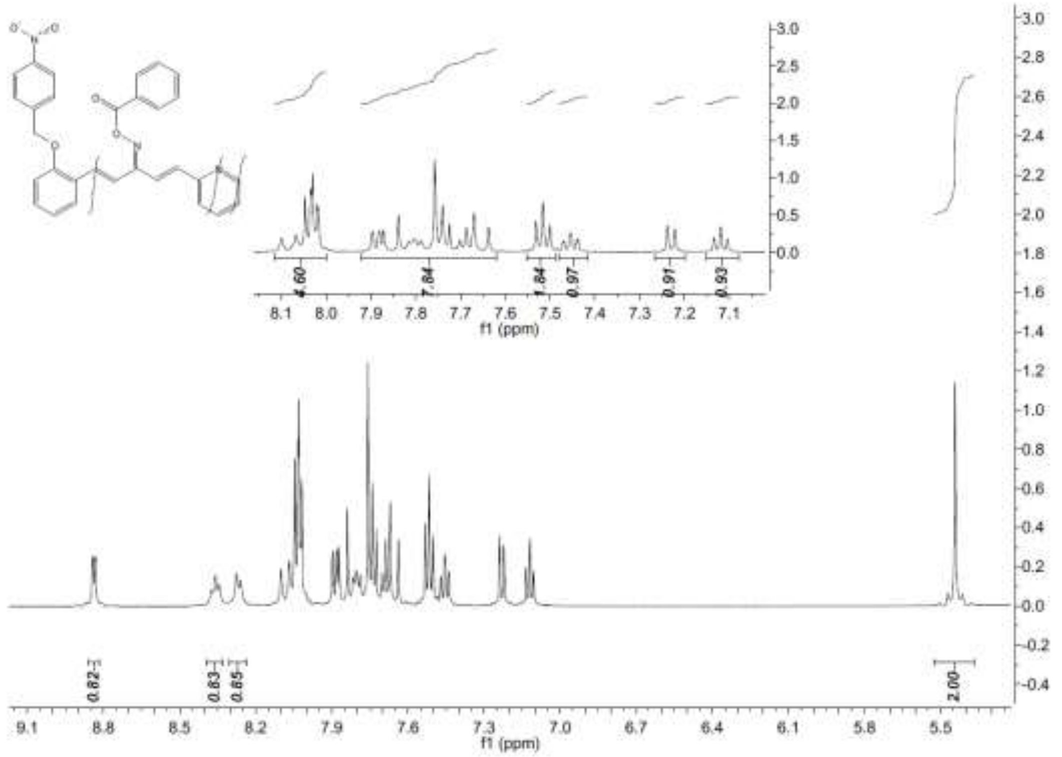
f1 (ppm)
4.60
7.84
1.84
0.97
0.91
0.93
0.82
0.83
0.85
2.00

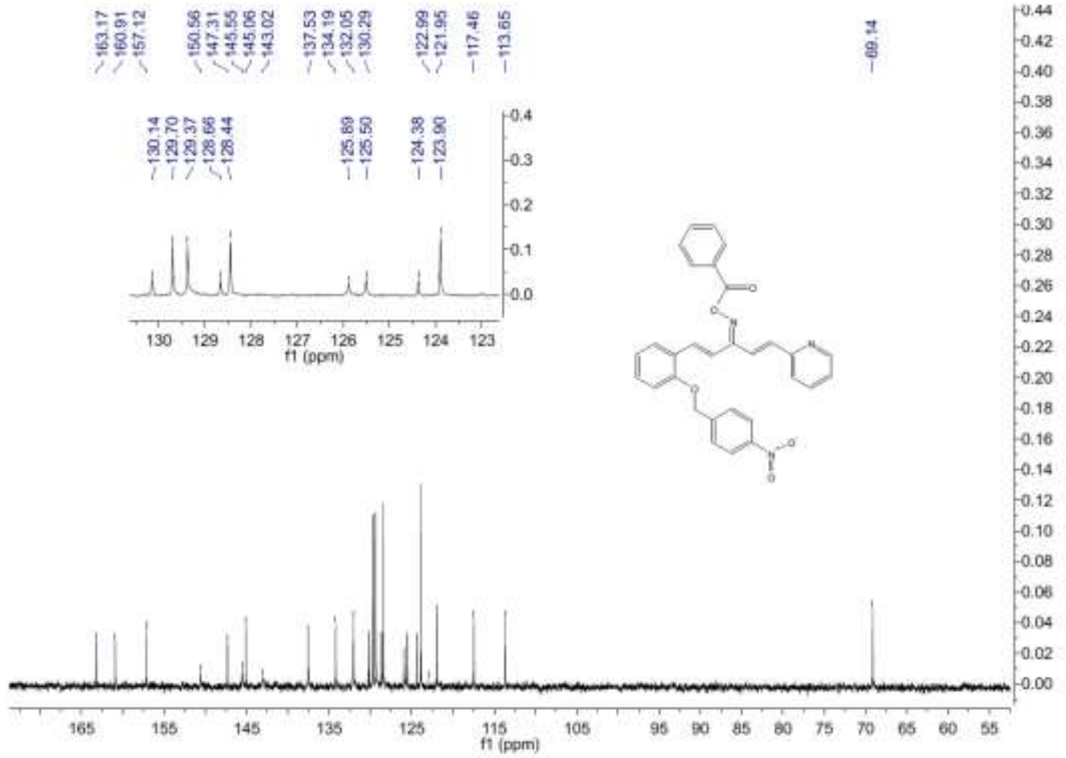
163.17
160.91
157.12
150.56
147.31
145.55
145.06
143.02
137.53
134.19
132.05
130.29
122.99
121.95
117.46
113.65
69.14
130.14
129.70
129.37
128.66
128.44
125.89
125.50
124.38
123.90
f1 (ppm)
f1 (ppm)

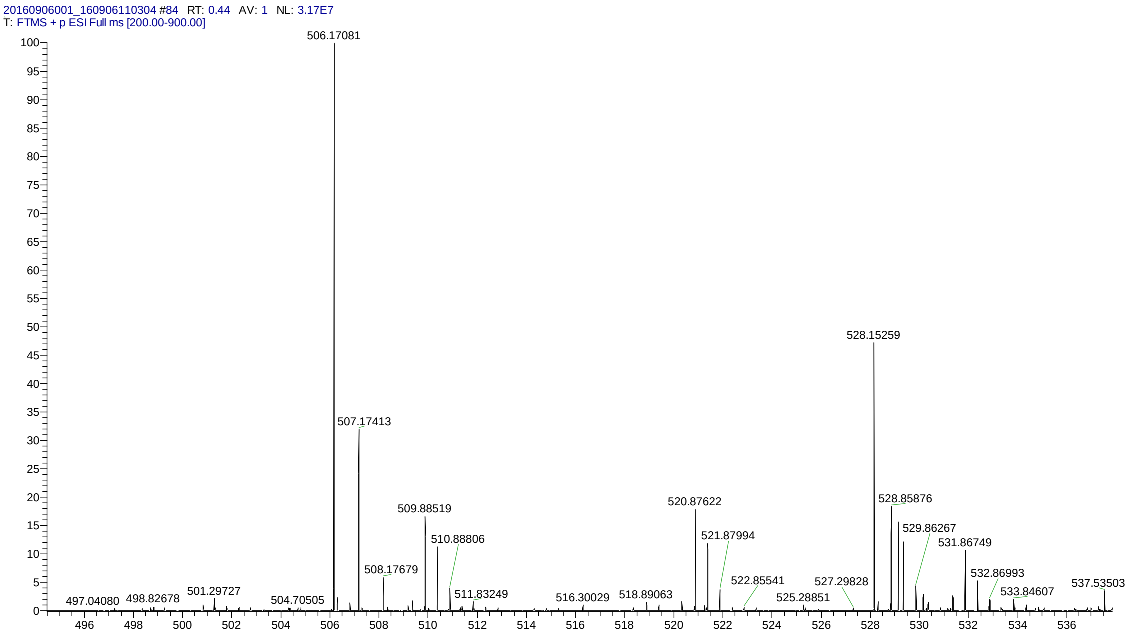
20160906001_160906110304 #84 RT: 0.44 AV: 1 NL: 3.17E7
T: FTMS + p ESI Full ms [200.00-900.00]
506.17081
507.17413
509.88519
510.88806
508.17679
528.15259
520.87622
521.87994
528.85876
529.86267
531.86749
532.86993
533.84607
537.53503
522.85541
527.29828
525.28851
497.04080
498.82678
501.29727
504.70505
511.83249
516.30029
518.89063
m/z

5c

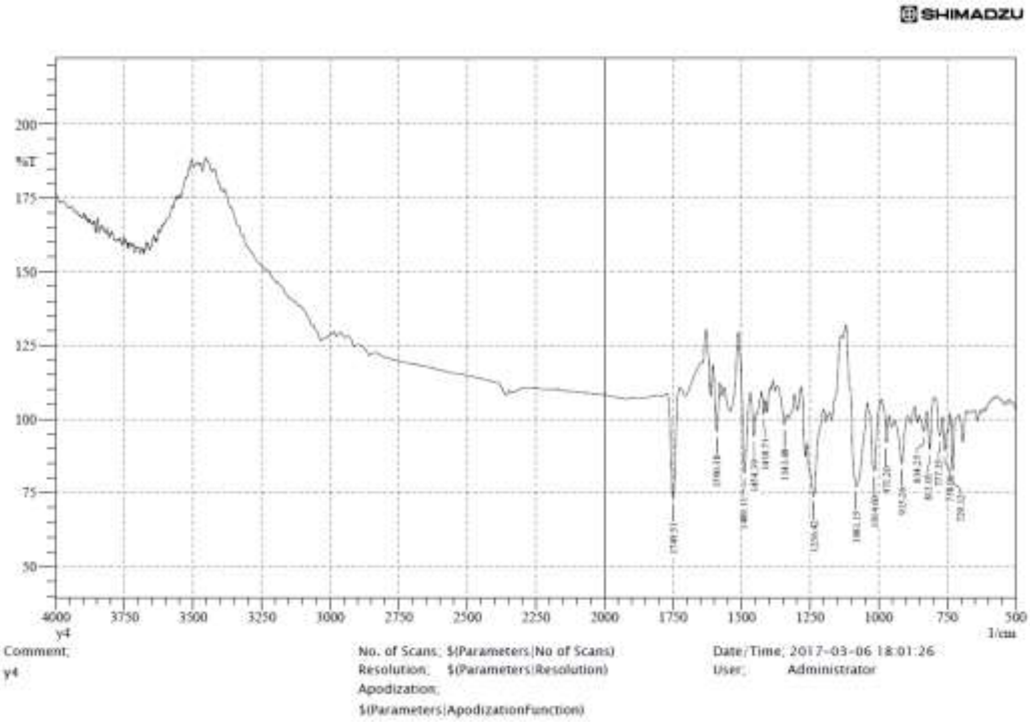
SHIMADZU
%T
1/cm
Comment;
y4
No. of Scans; $(Parameters|No of Scans)
Resolution; $(Parameters|Resolution)
Apodization;
$(Parameters|ApodizationFunction)
Date/Time; 2017-03-06 18:01:26
User; Administrator

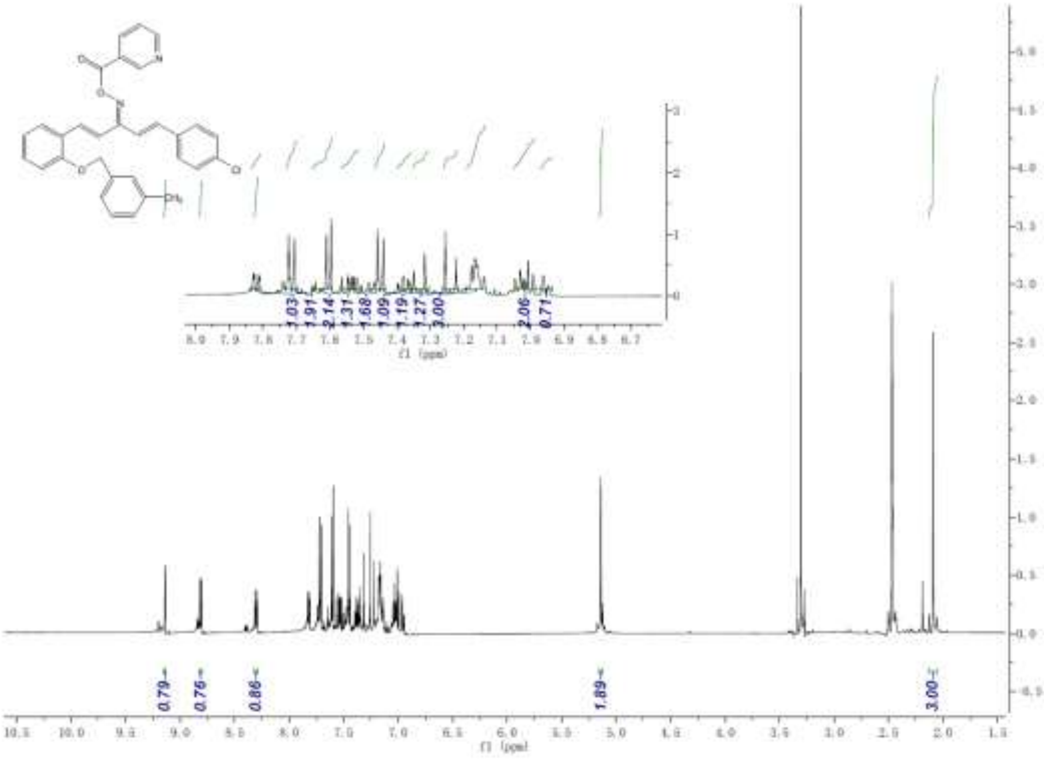
f1 (ppm)

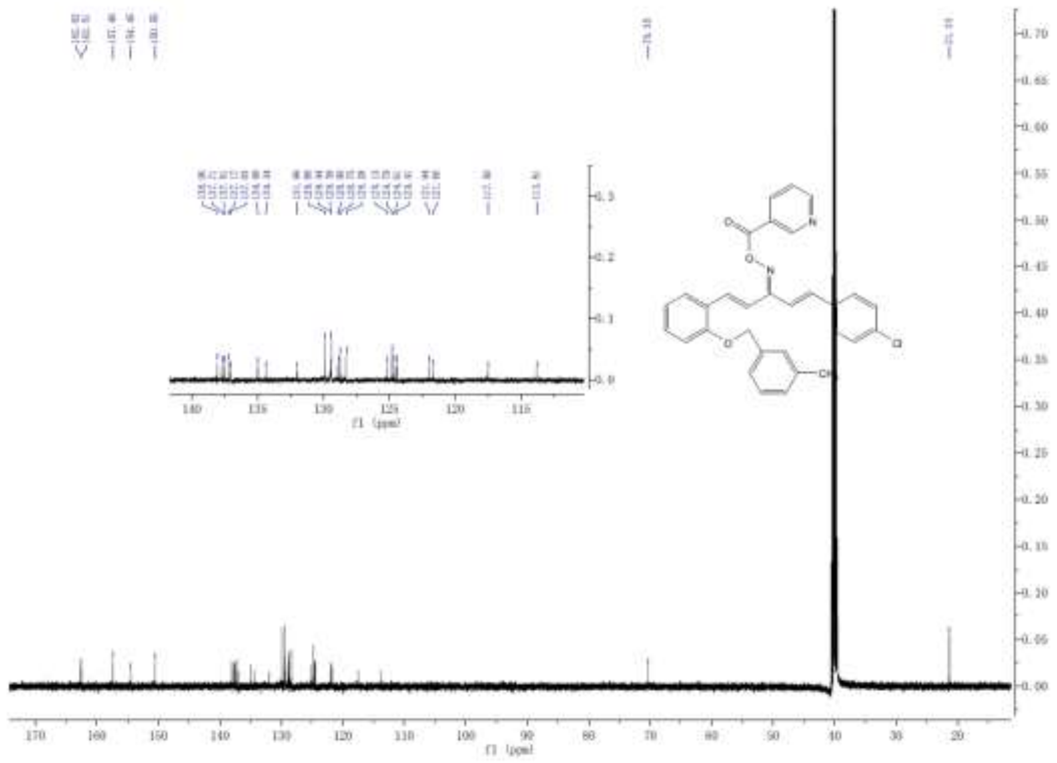

20160906002_160906110757 #148 RT: 0.78 AV: 1 NL: 9.99E6
T: FTMS + p ESI Full ms [200.00-900.00]

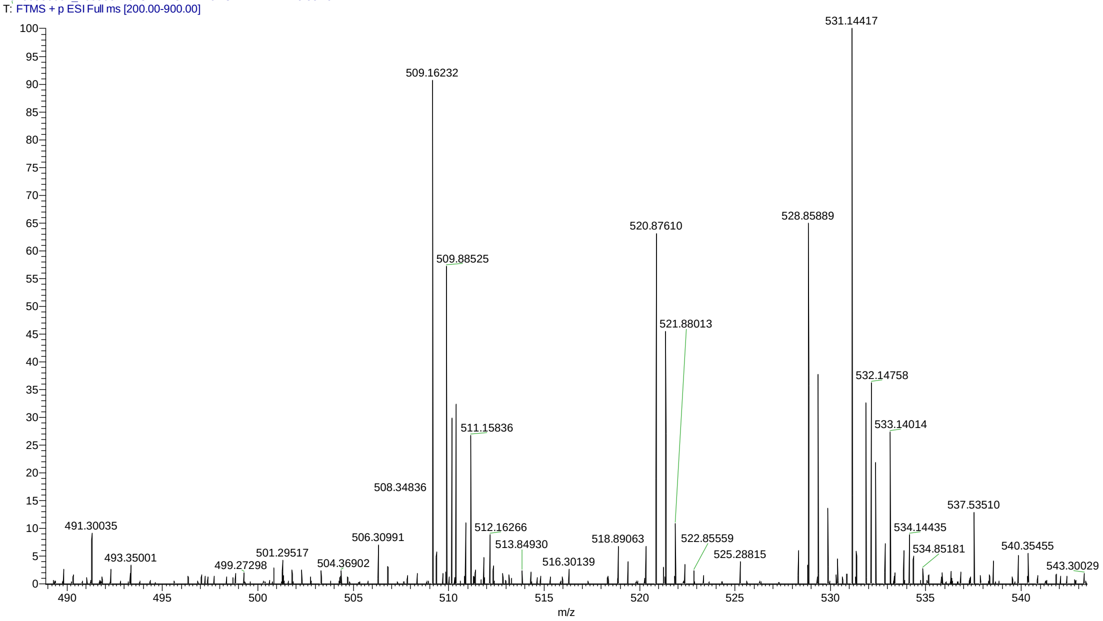

5d

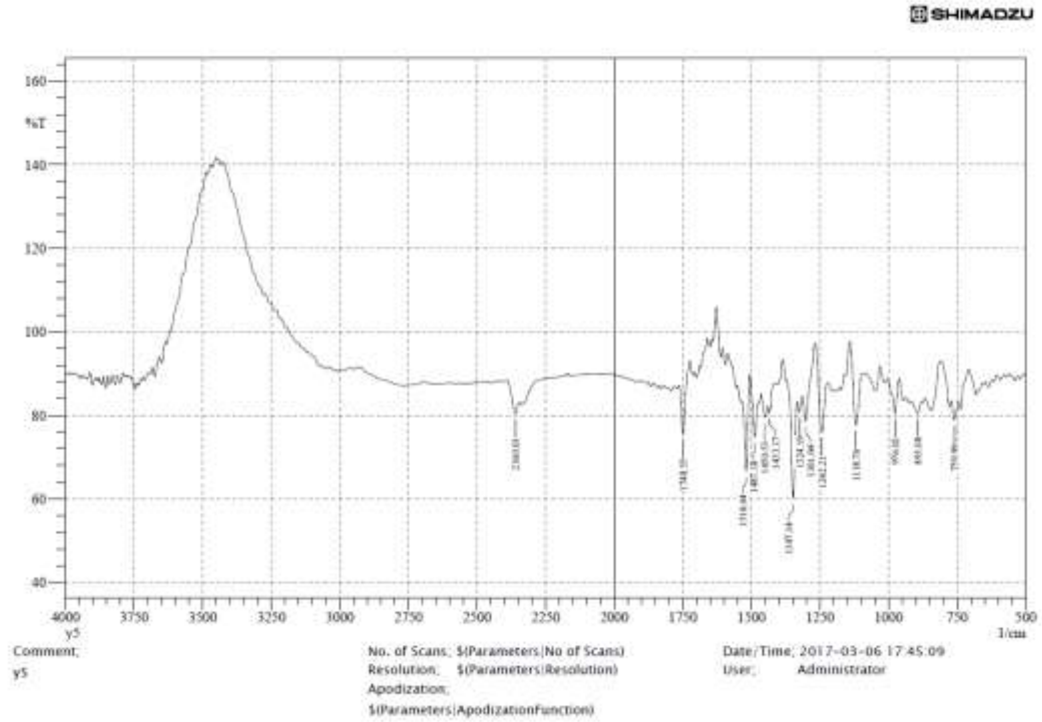
SHIMADZU
%T
160
140
120
100
80
60
40
4000
3750
3500
3250
3000
2750
2500
2250
2000
1750
1500
1250
1000
750
500
1/cm
y5
Comment;
y5
No. of Scans; $(Parameters|No of Scans)
Resolution; $(Parameters|Resolution)
Apodization;
$(Parameters|ApodizationFunction)
Date/Time; 2017-03-06 17:45:09
User; Administrator

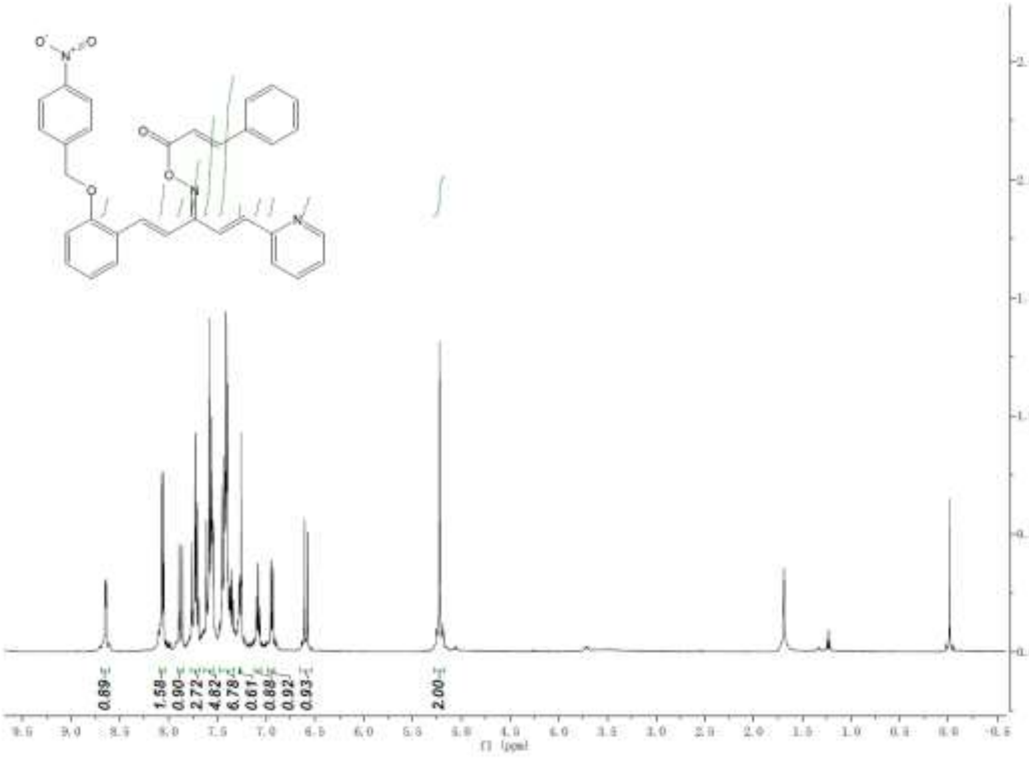
0.89
1.58
0.90
2.72
4.82
6.78
0.61
0.88
0.92
0.93
2.00
f1 (ppm)

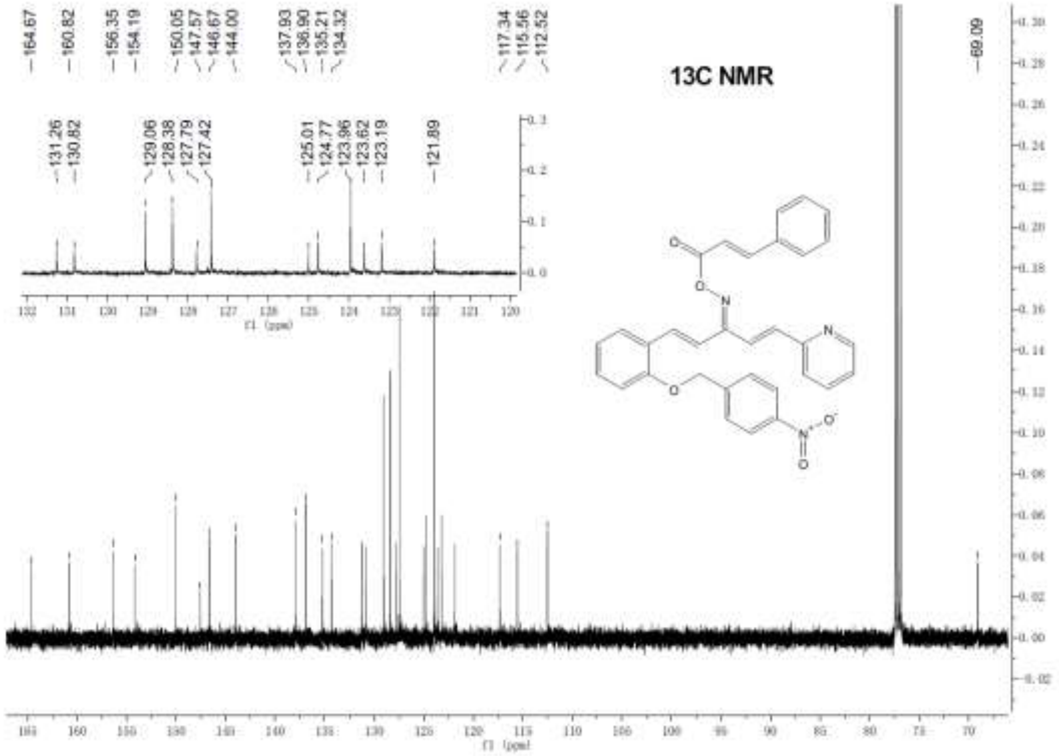
13C NMR
164.67
160.82
156.35
154.19
150.05
147.57
146.67
144.00
137.93
136.90
135.21
134.32
117.34
115.56
112.52
69.09
131.26
130.82
129.06
128.38
127.79
127.42
125.01
124.77
123.96
123.62
123.19
121.89
f1 (ppm)

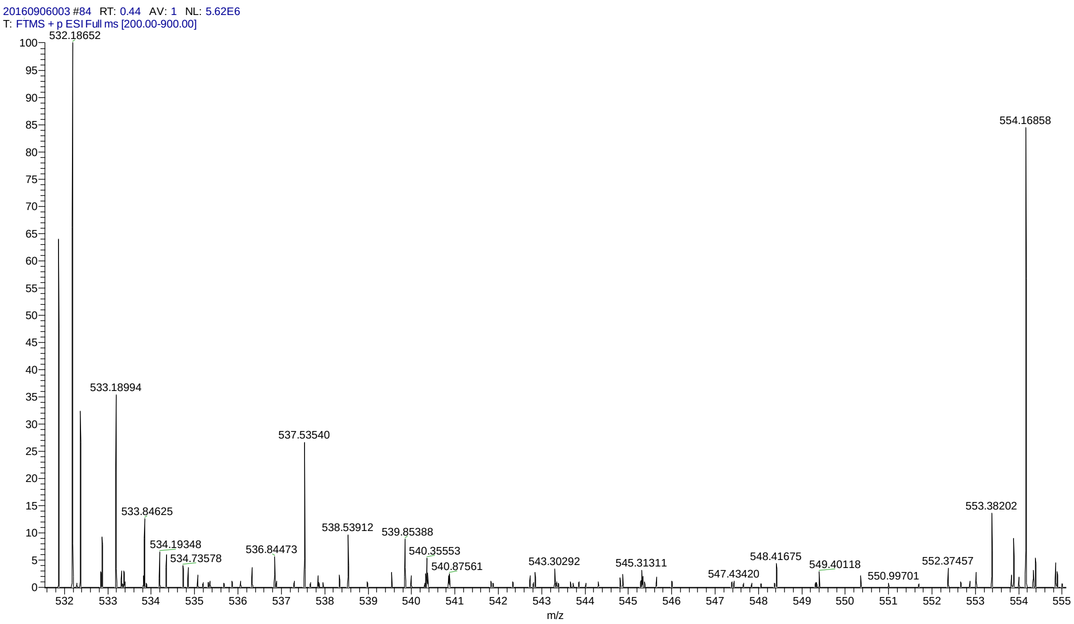
20160906003 #84 RT: 0.44 AV: 1 NL: 5.62E6
T: FTMS + p ESI Full ms [200.00-900.00]
532.18652
554.16858
533.18994
537.53540
553.38202
533.84625
538.53912
539.85388
534.19348
534.73578
536.84473
540.35553
540.87561
543.30292
545.31311
548.41675
547.43420
549.40118
550.99701
552.37457
m/z

5e

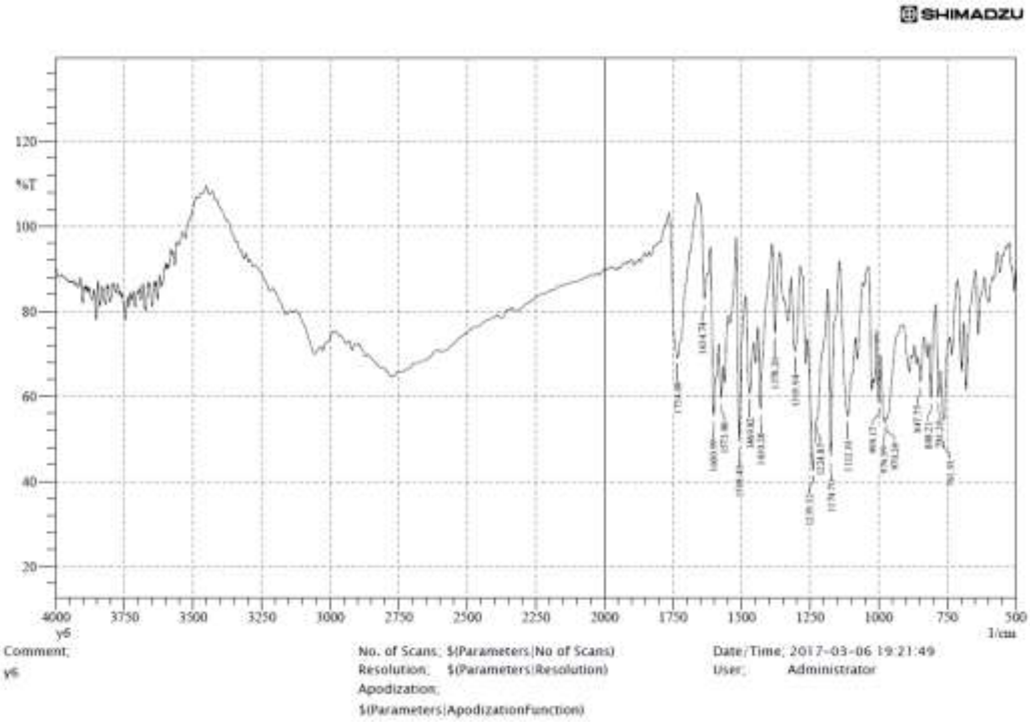
SHIMADZU
%T
1/cm
y6
Comment;
y6
No. of Scans; $(Parameters|No of Scans)
Resolution; $(Parameters|Resolution)
Apodization;
$(Parameters|ApodizationFunction)
Date/Time; 2017-03-06 19:21:49
User; Administrator

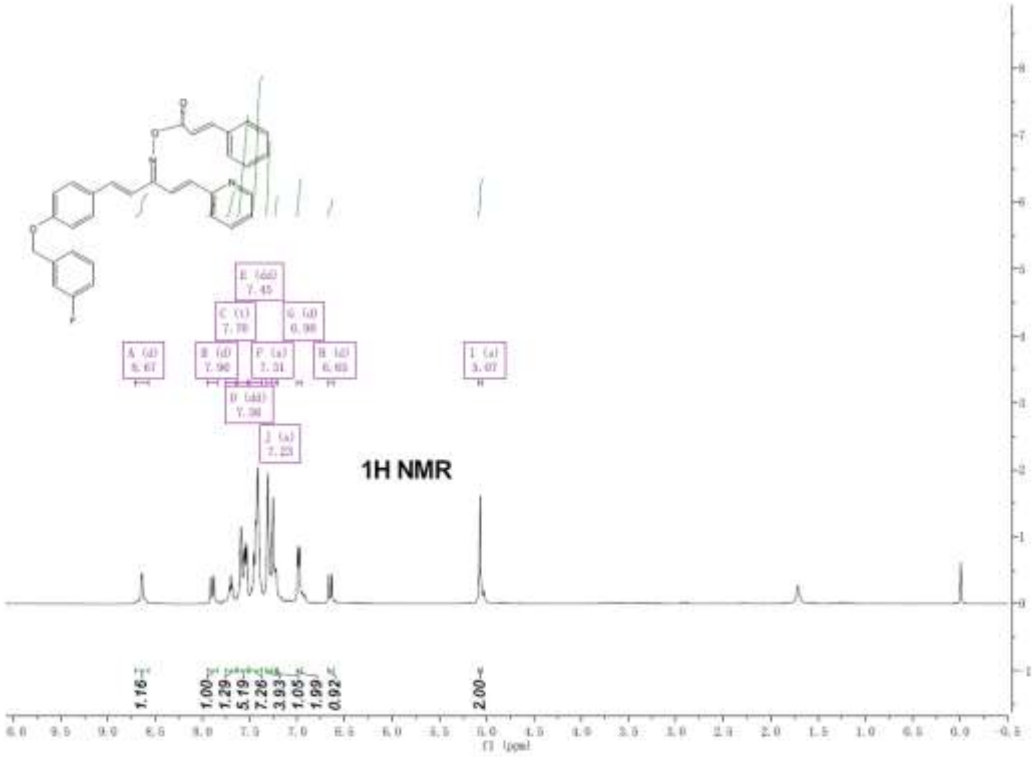
1H NMR
f1 (ppm)

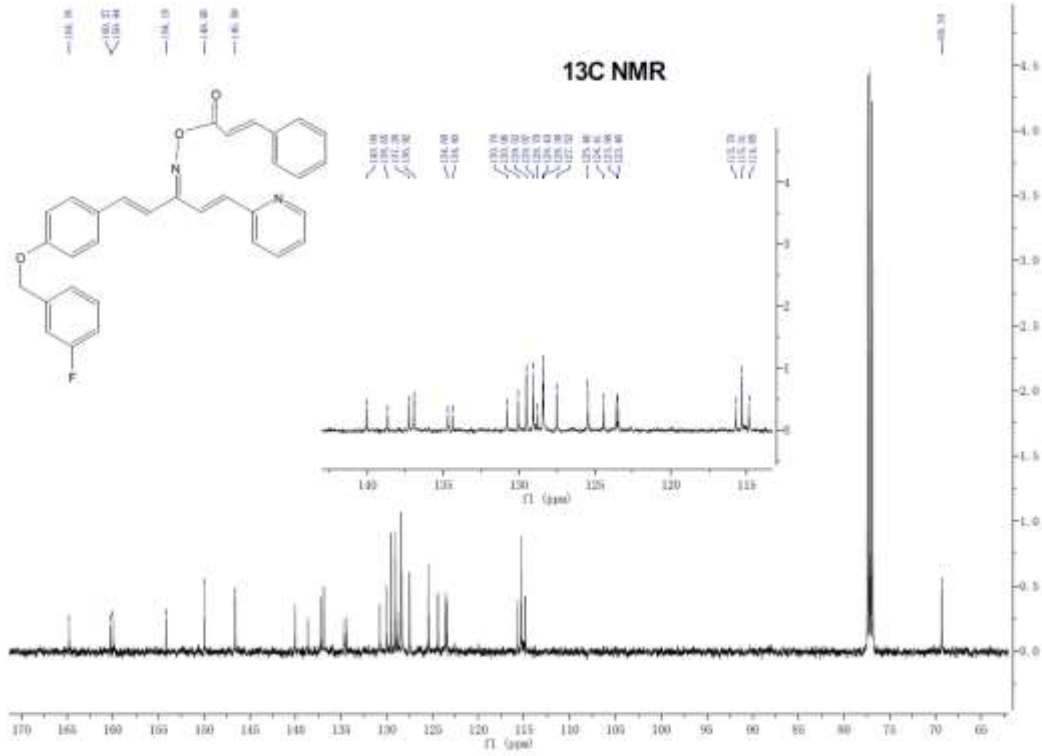
13C NMR
f1 (ppm)

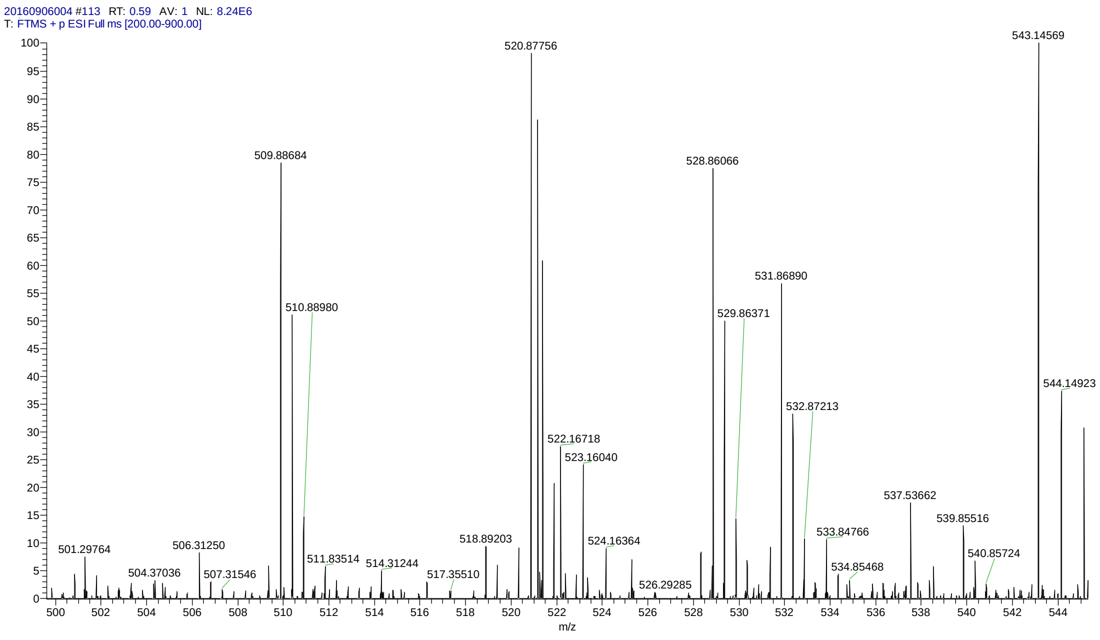
20160906004 #113 RT: 0.59 AV: 1 NL: 8.24E6
T: FTMS + p ESI Full ms [200.00-900.00]
543.14569
520.87756
509.88684
528.86066
531.86890
510.88980
529.86371
544.14923
532.87213
522.16718
523.16040
537.53662
539.85516
533.84766
518.89203
524.16364
540.85724
501.29764
506.31250
534.85468
511.83514
514.31244
504.37036
507.31546
517.35510
526.29285
m/z

5f

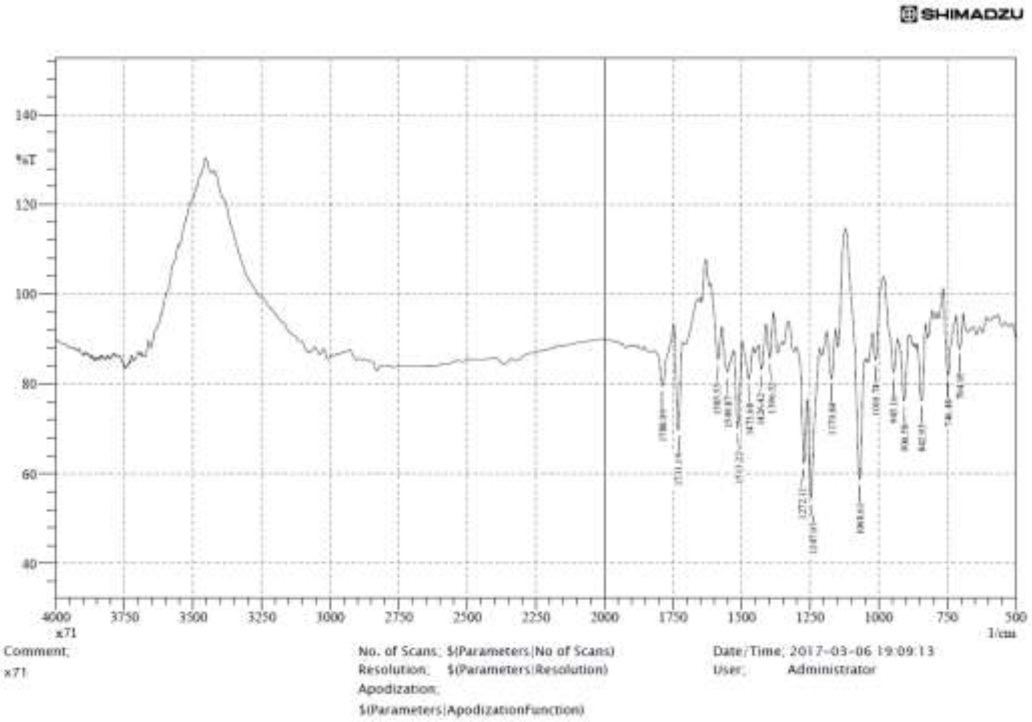
SHIMADZU
%T
1/cm
Comment;
x71
No. of Scans; $(Parameters|No of Scans)
Resolution; $(Parameters|Resolution)
Apodization;
$(Parameters|ApodizationFunction)
Date/Time; 2017-03-06 19:09:13
User; Administrator

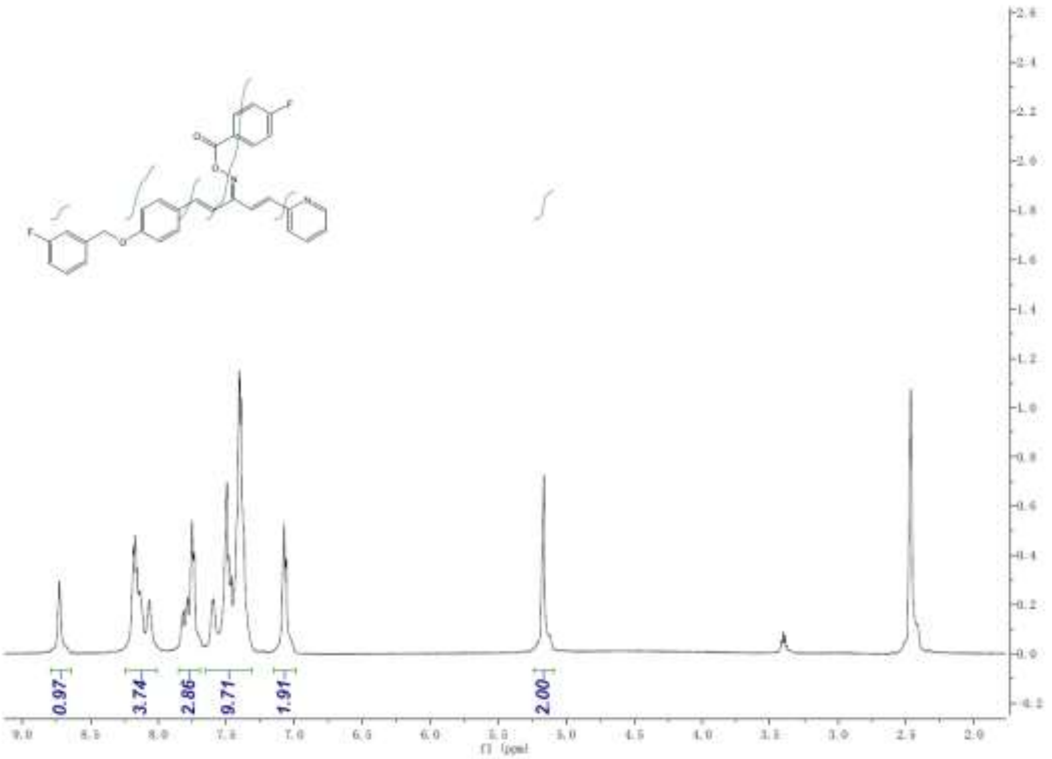
0.97
3.74
2.86
9.71
1.91
2.00
f1 (ppm)

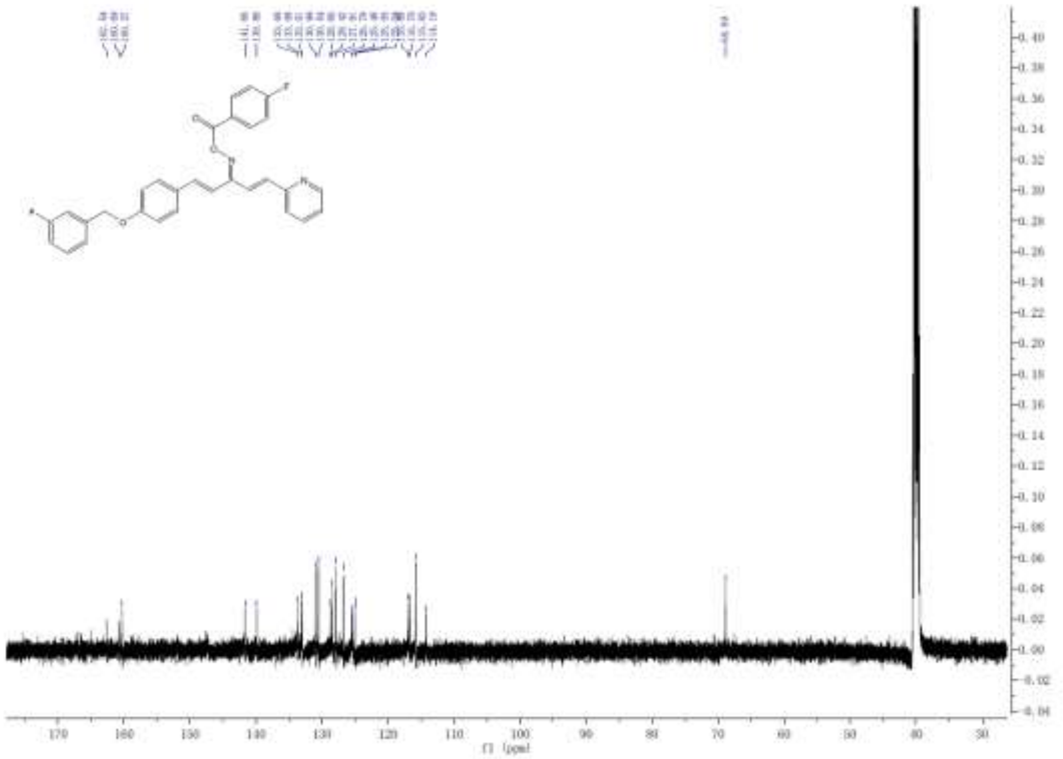

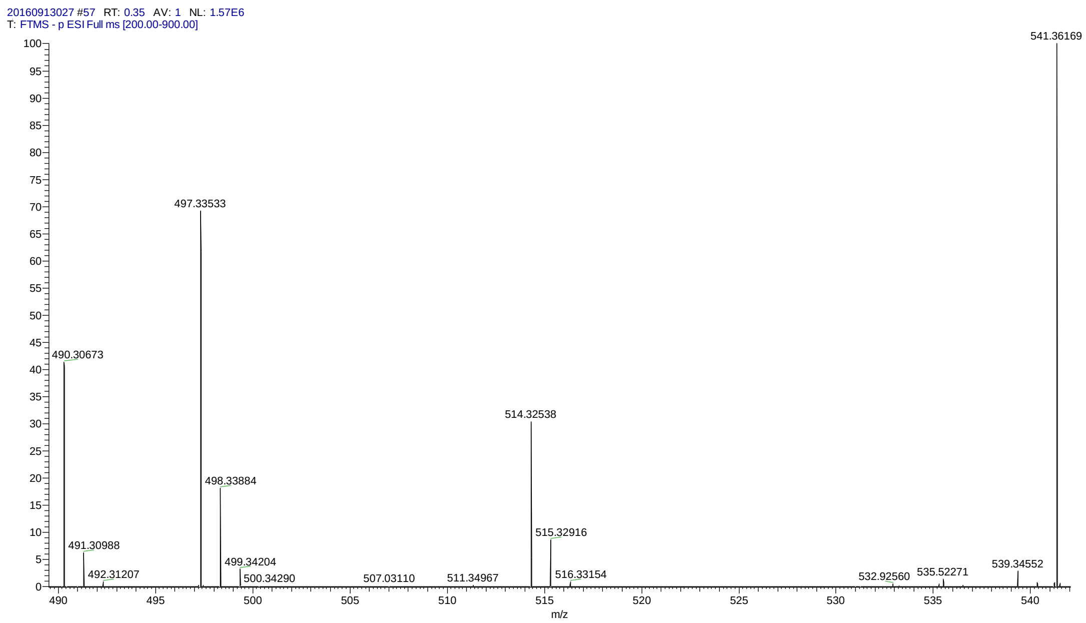

20160913027 #57 RT: 0.35 AV: 1 NL: 1.57E6
T: FTMS - p ESI Full ms [200.00-900.00]
541.36169
497.33533
490.30673
514.32538
498.33884
515.32916
491.30988
499.34204
492.31207
500.34290
507.03110
511.34967
516.33154
532.92560
535.52271
539.34552
m/z

5g

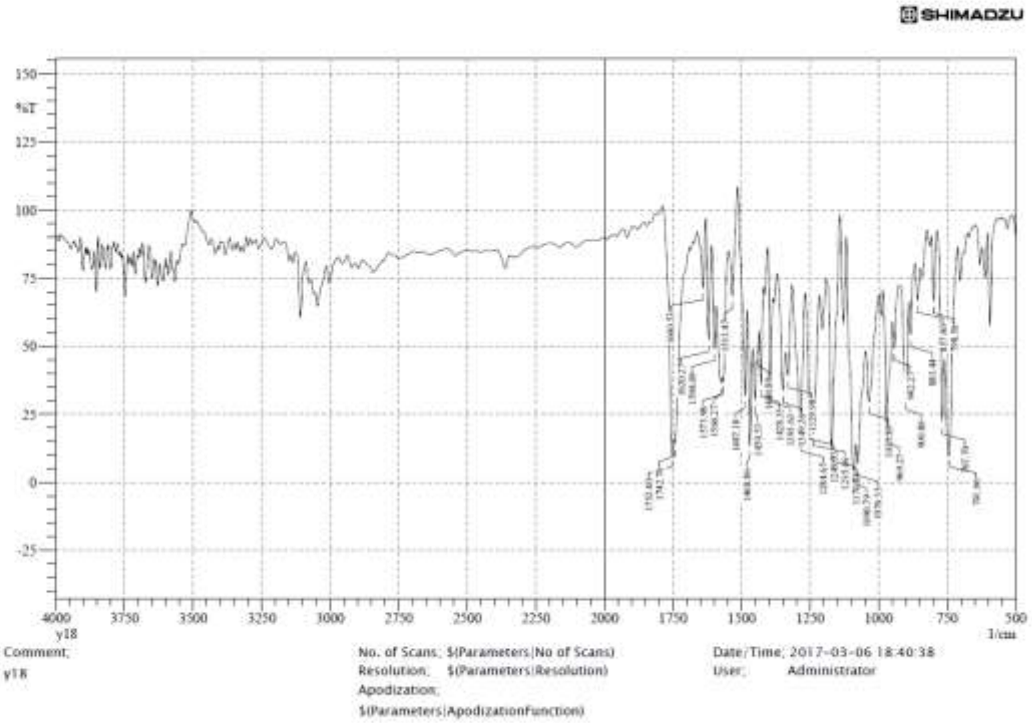
SHIMADZU
%T
1/cm
y18
Comment;
y18
No. of Scans; $(Parameters|No of Scans)
Resolution; $(Parameters|Resolution)
Apodization;
$(Parameters|ApodizationFunction)
Date/Time; 2017-03-06 18:40:38
User; Administrator

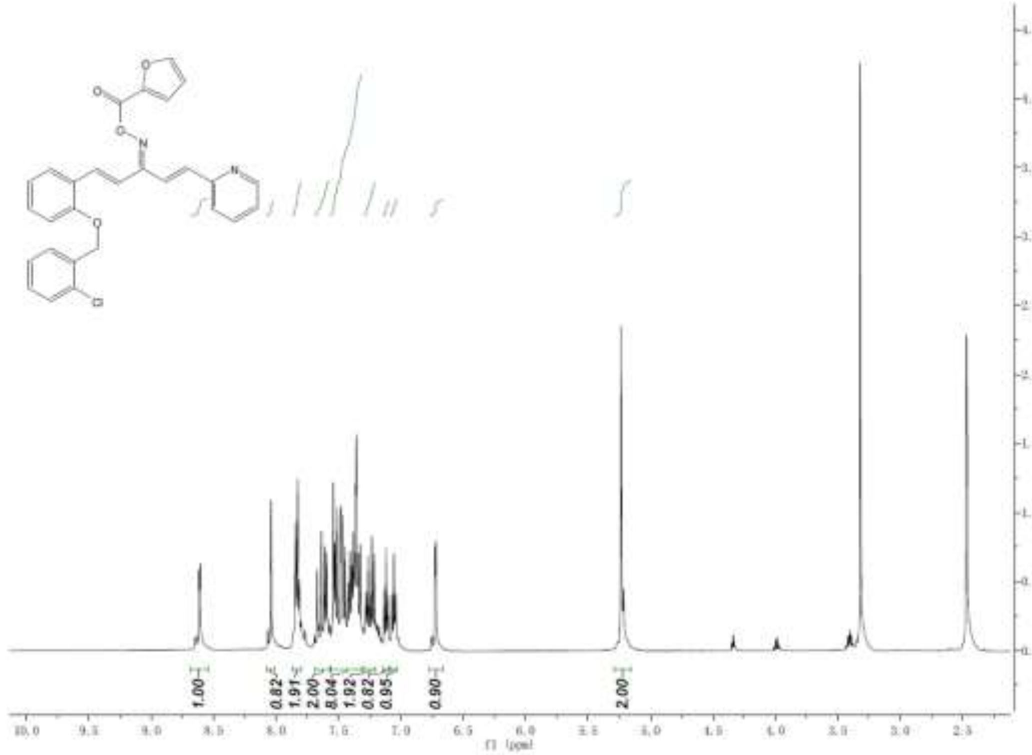
1.00
0.82
1.91
2.00
8.04
1.92
0.82
0.95
0.90
2.00
f1 (ppm)

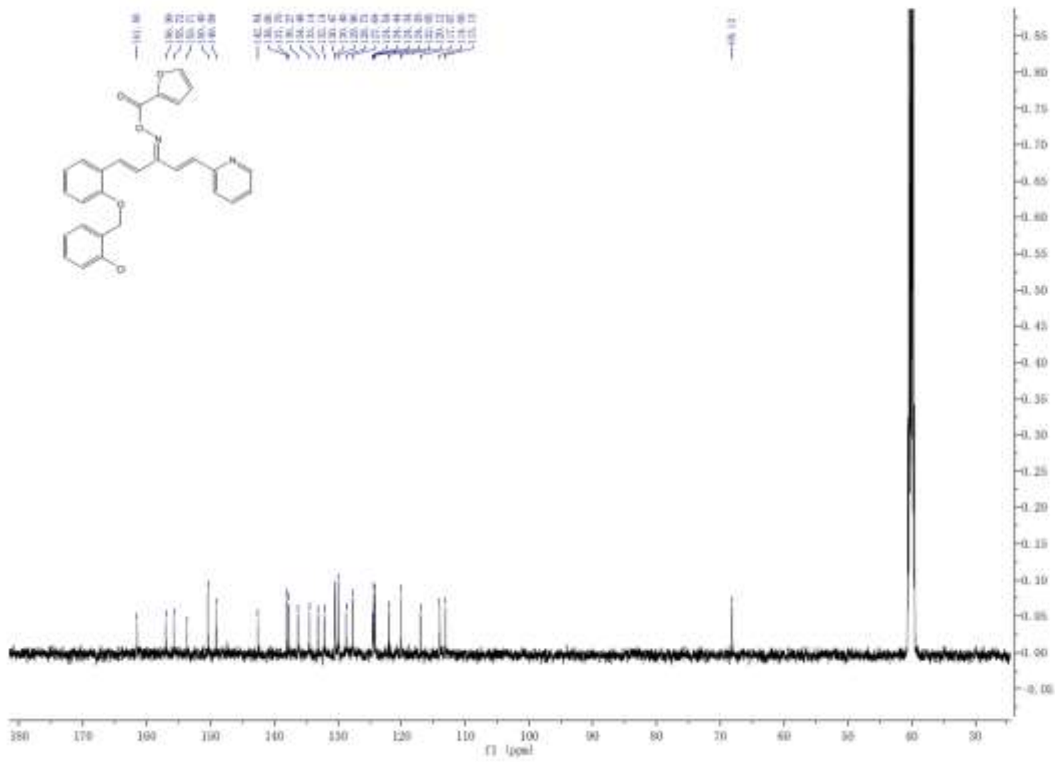
f1 (ppm)

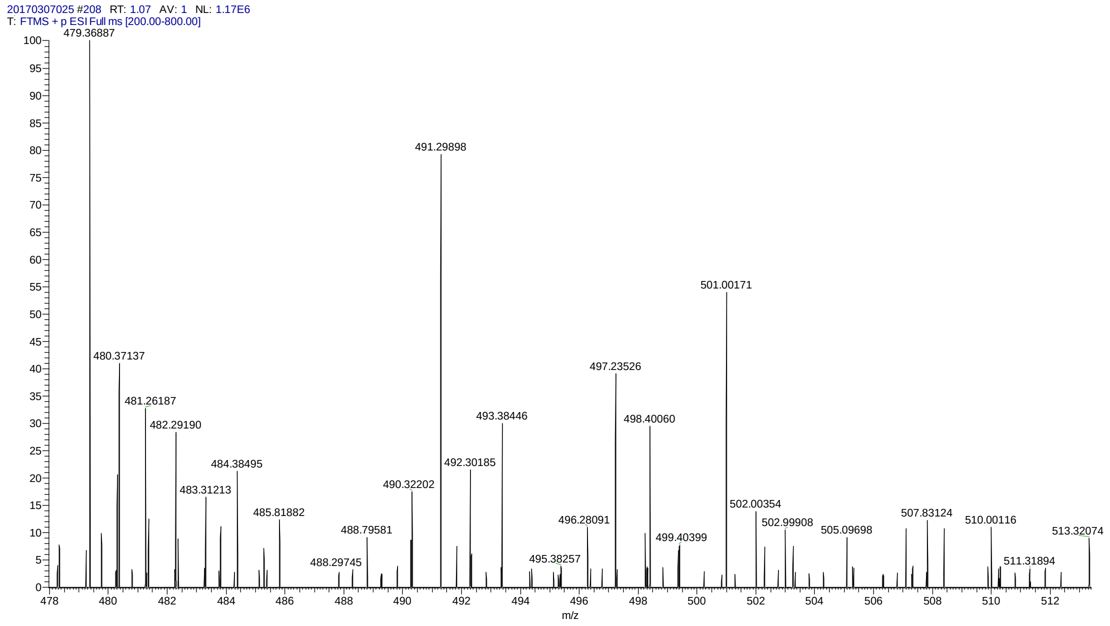
20170307025 #208 RT: 1.07 AV: 1 NL: 1.17E6
T: FTMS + p ESI Full ms [200.00-800.00]
479.36887
491.29898
501.00171
480.37137
497.23526
481.26187
493.38446
482.29190
498.40060
484.38495
492.30185
483.31213
490.32202
485.81882
502.00354
507.83124
510.00116
496.28091
502.99908
488.79581
505.09698
513.32074
499.40399
488.29745
495.38257
511.31894
m/z

5h

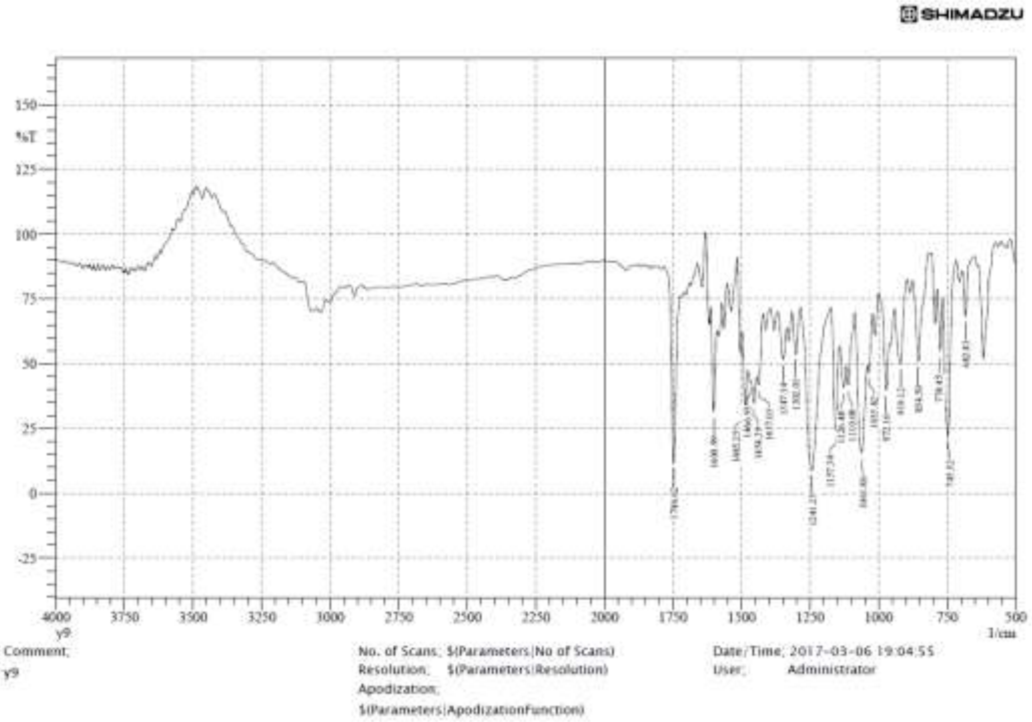
SHIMADZU
%T
1/cm
y9
Comment;
y9
No. of Scans; $(Parameters|No of Scans)
Resolution; $(Parameters|Resolution)
Apodization;
$(Parameters|ApodizationFunction)
Date/Time; 2017-03-06 19:04:55
User; Administrator

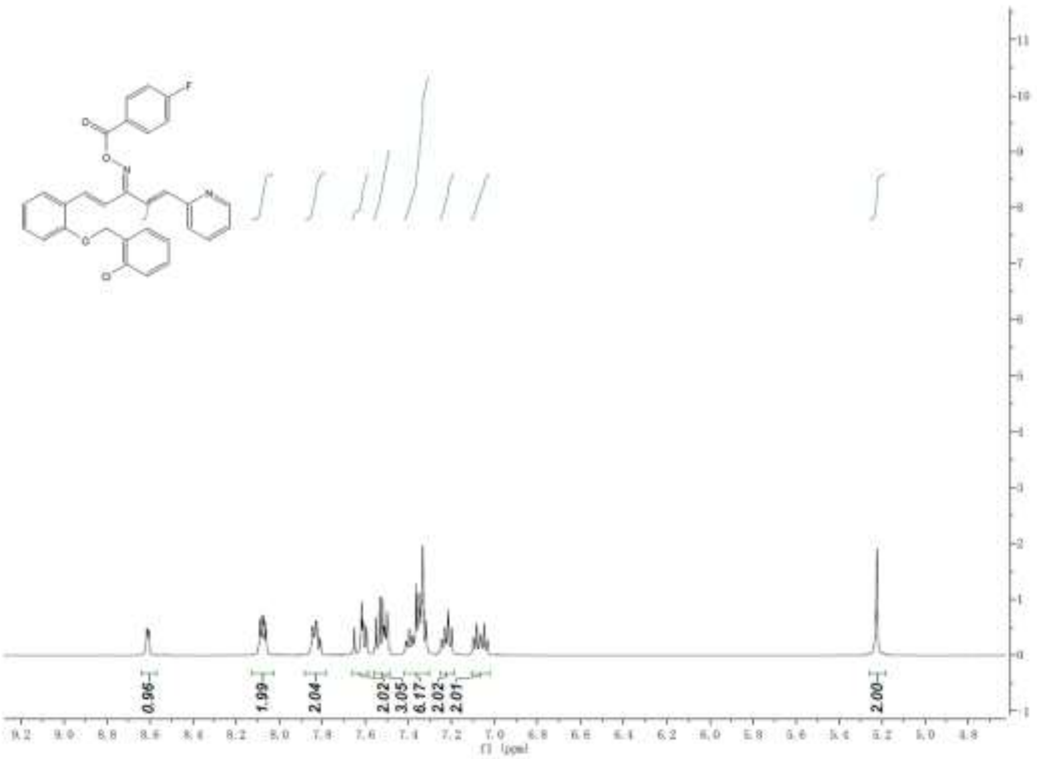
F
O
O
N
N
O
Cl
0.96
1.99
2.04
2.02
3.05
6.17
2.02
2.01
2.00
f1 (ppm)

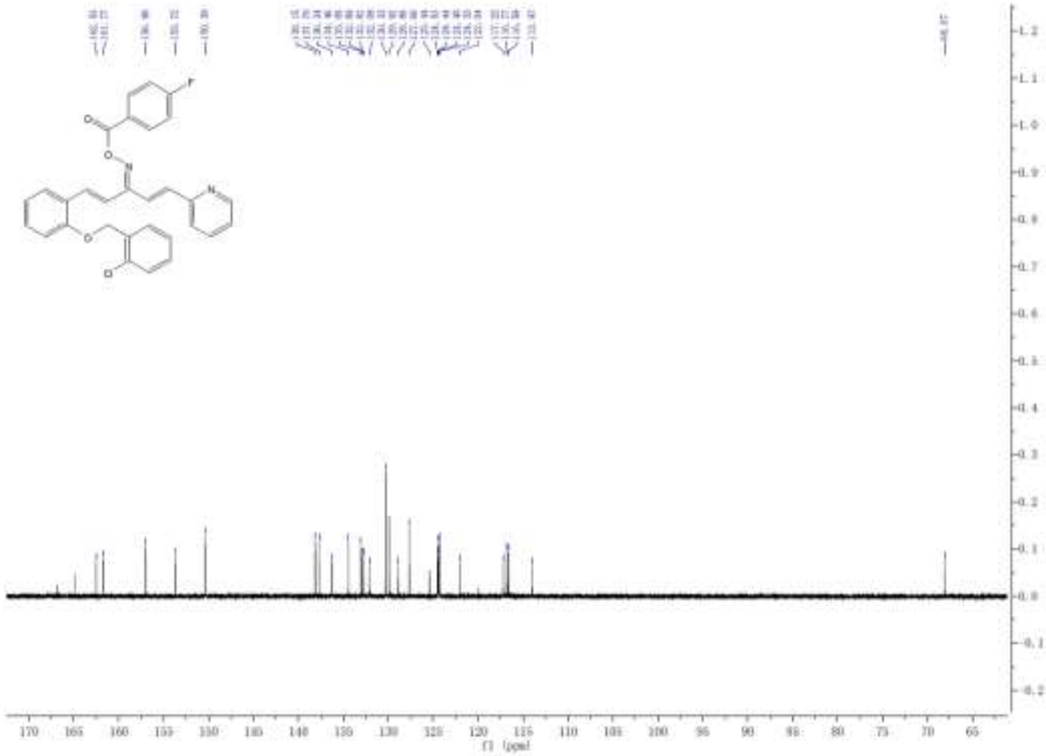
f1 (ppm)
F
O
N
Cl

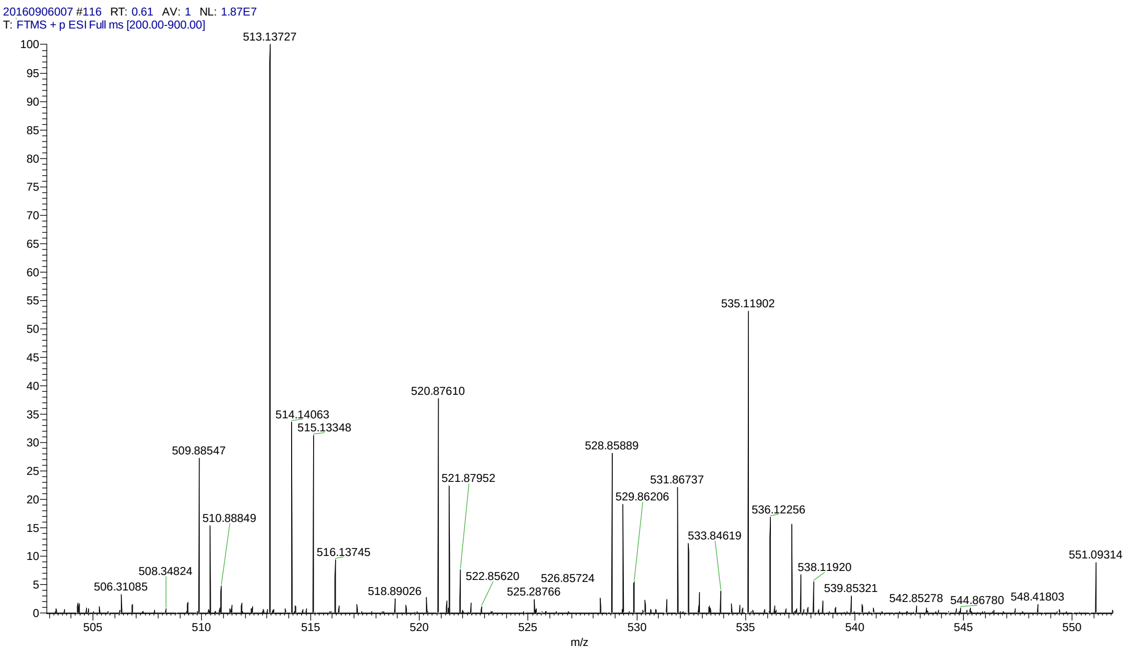
20160906007 #116 RT: 0.61 AV: 1 NL: 1.87E7
T: FTMS + p ESI Full ms [200.00-900.00]
513.13727
535.11902
520.87610
514.14063
515.13348
509.88547
528.85889
521.87952
531.86737
529.86206
536.12256
510.88849
533.84619
516.13745
538.11920
508.34824
551.09314
506.31085
522.85620
526.85724
518.89026
525.28766
539.85321
542.85278
544.86780
548.41803
m/z

5i

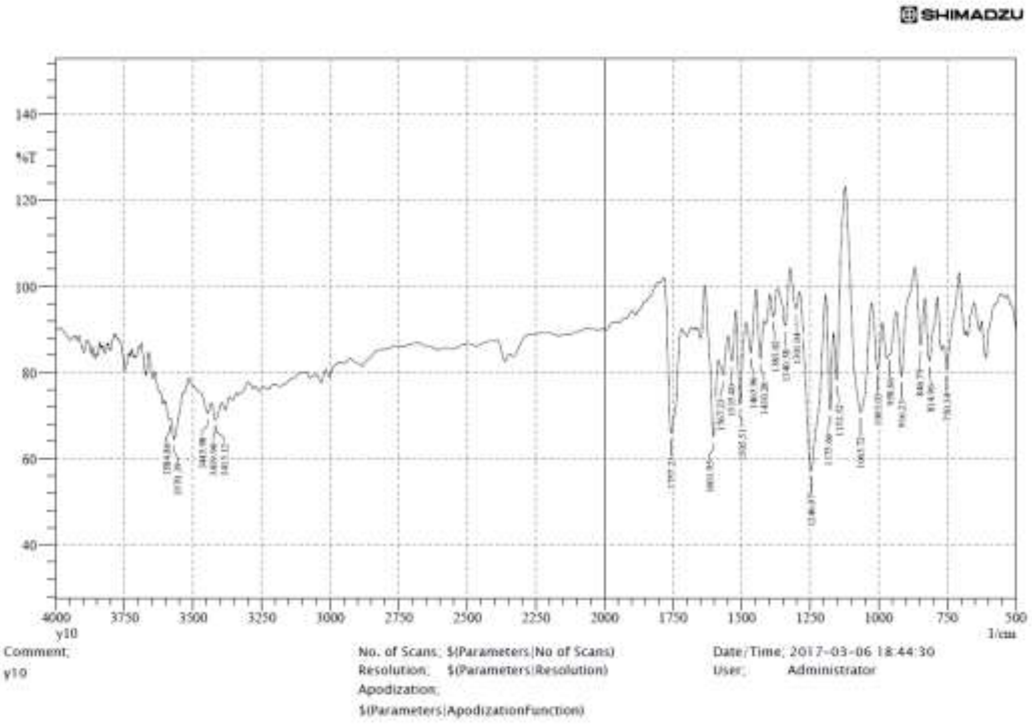

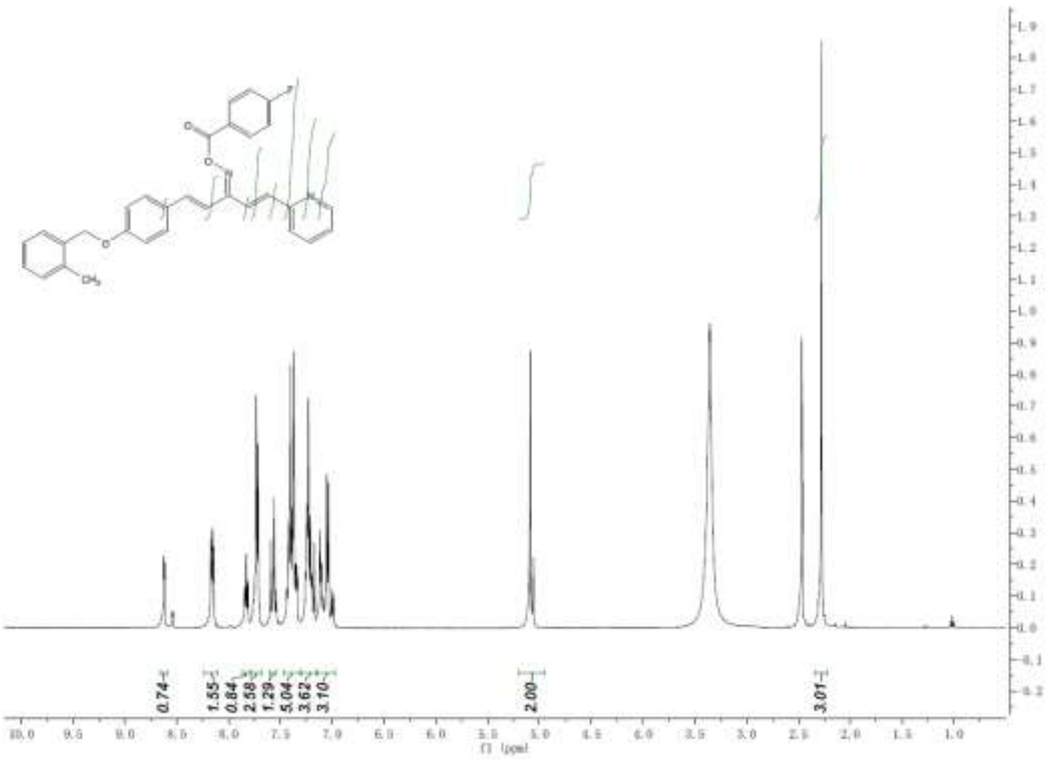

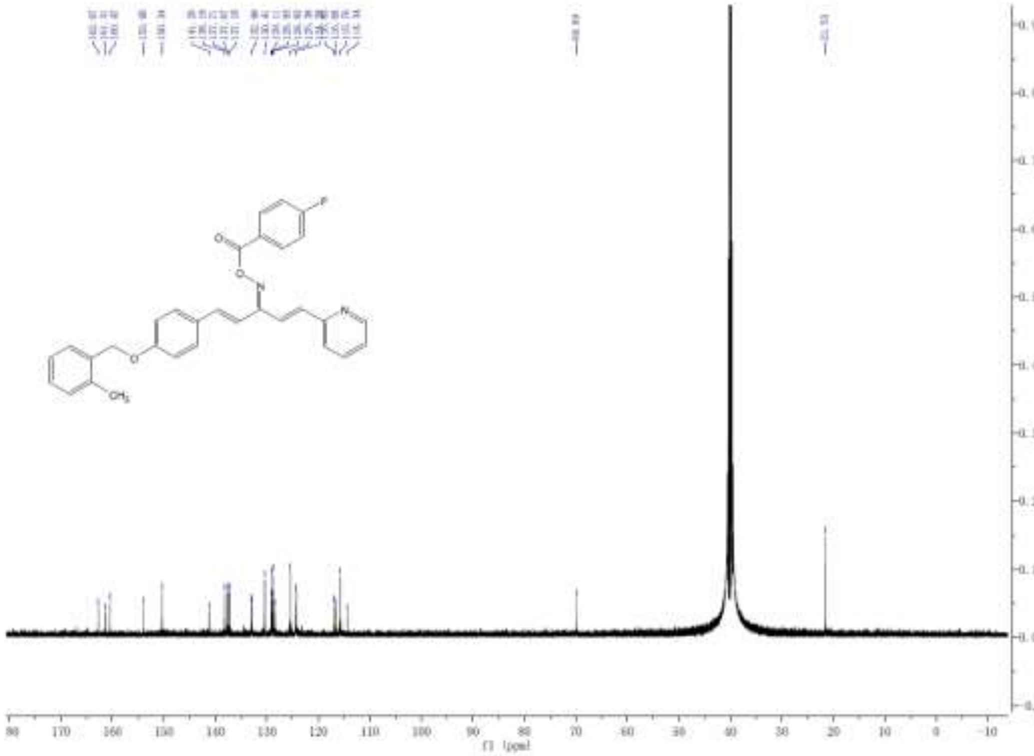

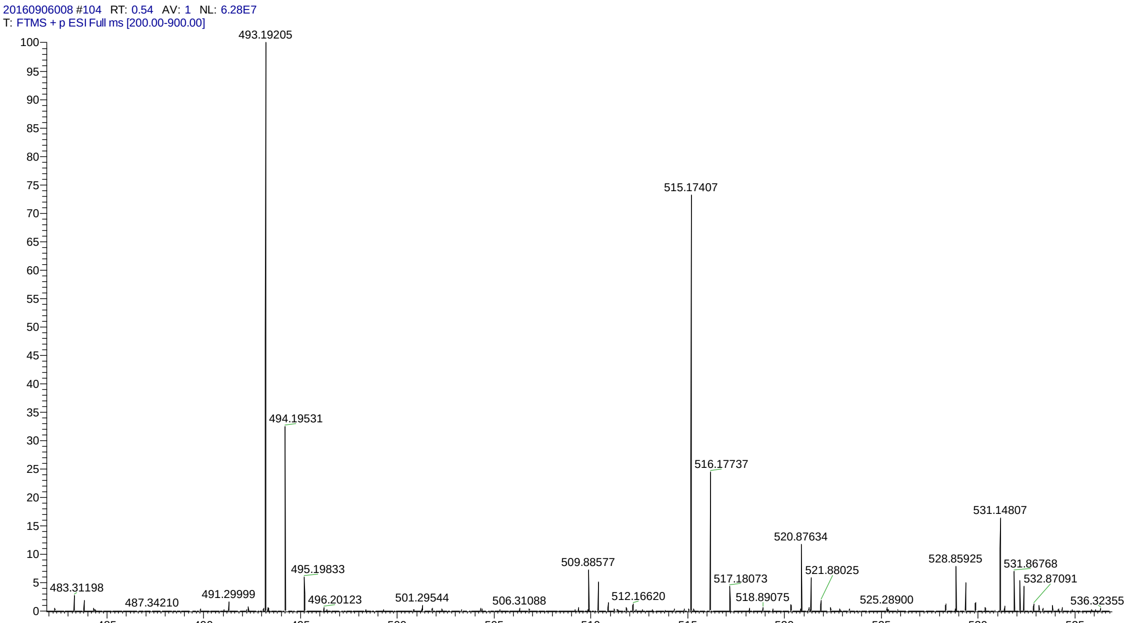
20160906008 #104 RT: 0.54 AV: 1 NL: 6.28E7
T: FTMS + p ESI Full ms [200.00-900.00]
493.19205
515.17407
494.19531
516.17737
531.14807
520.87634
528.85925
531.86768
532.87091
509.88577
495.19833
521.88025
517.18073
483.31198
491.29999
496.20123
501.29544
506.31088
512.16620
518.89075
525.28900
536.32355
487.34210
m/z

5j

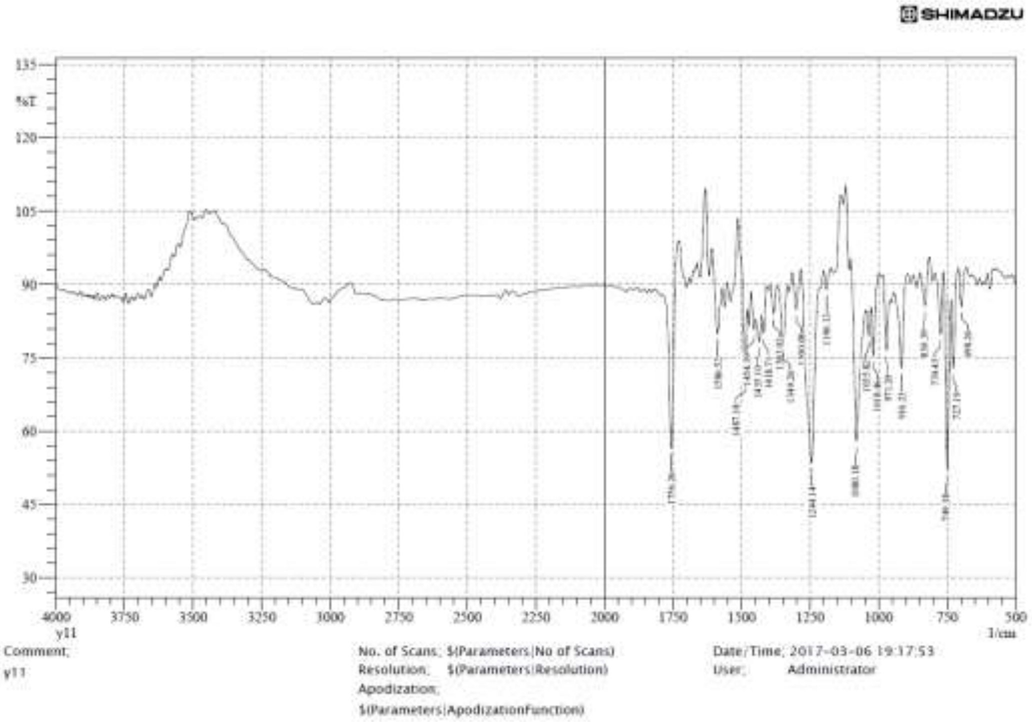

SHIMADZU
%T
1/cm
Comment;
y11
No. of Scans; $(Parameters|No of Scans)
Resolution; $(Parameters|Resolution)
Apodization;
$(Parameters|ApodizationFunction)
Date/Time; 2017-03-06 19:17:53
User; Administrator

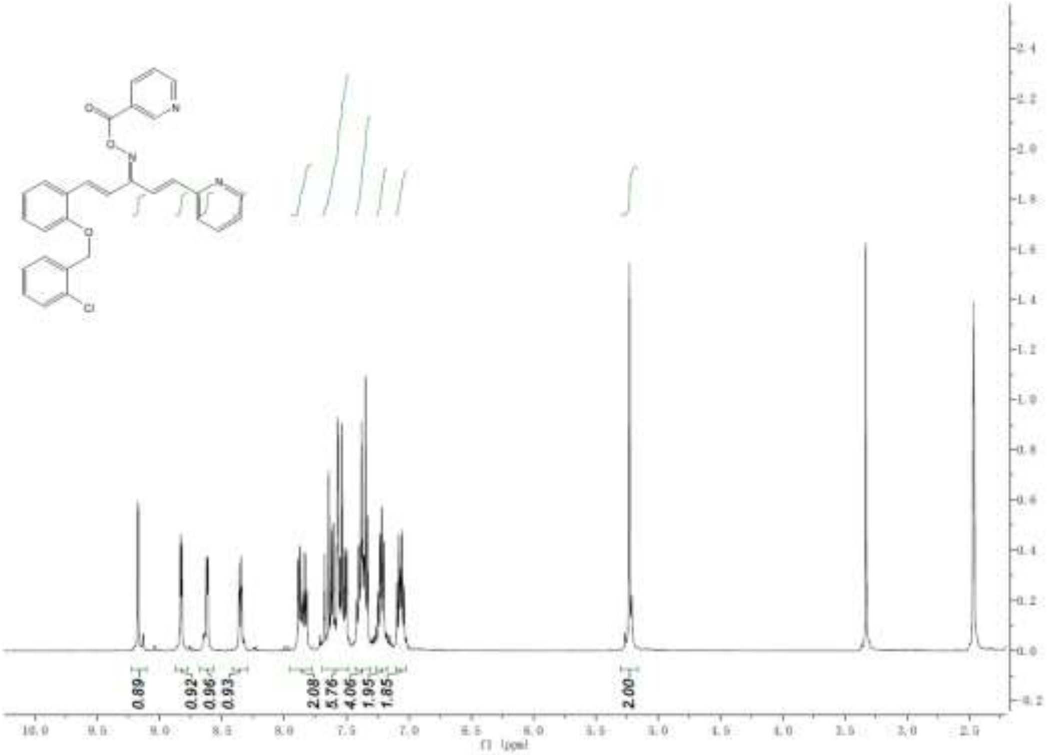

0.89
0.92
0.96
0.93
2.08
5.76
4.06
1.95
1.85
2.00
f1 (ppm)

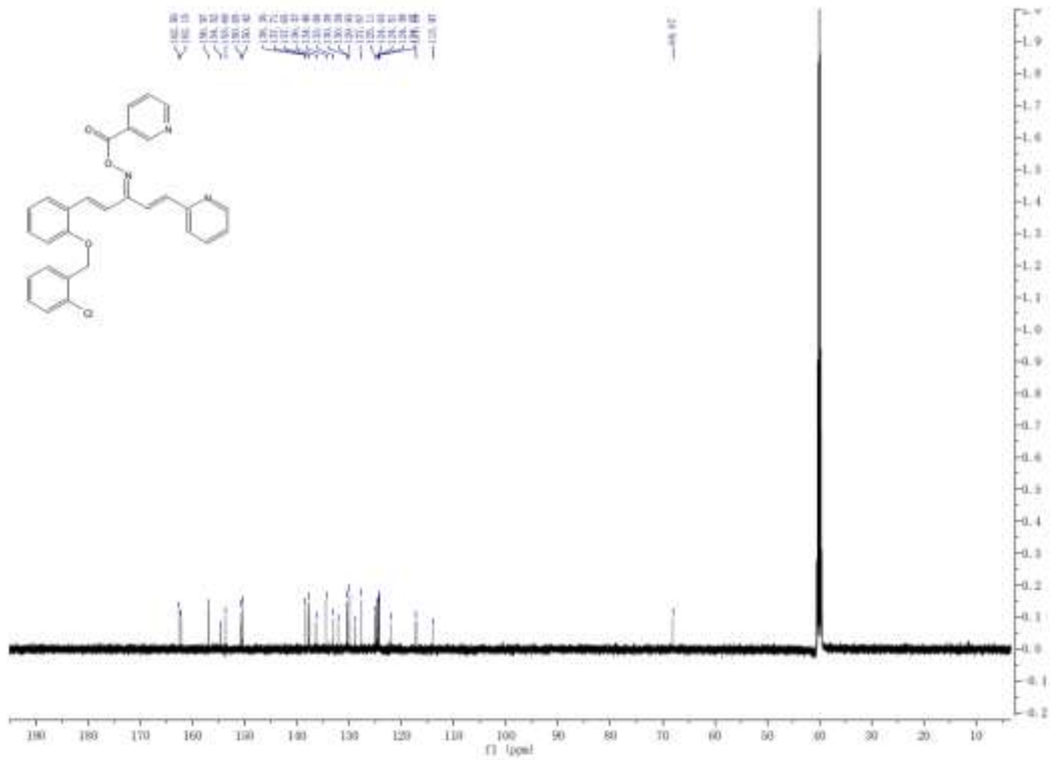
f1 (ppm)

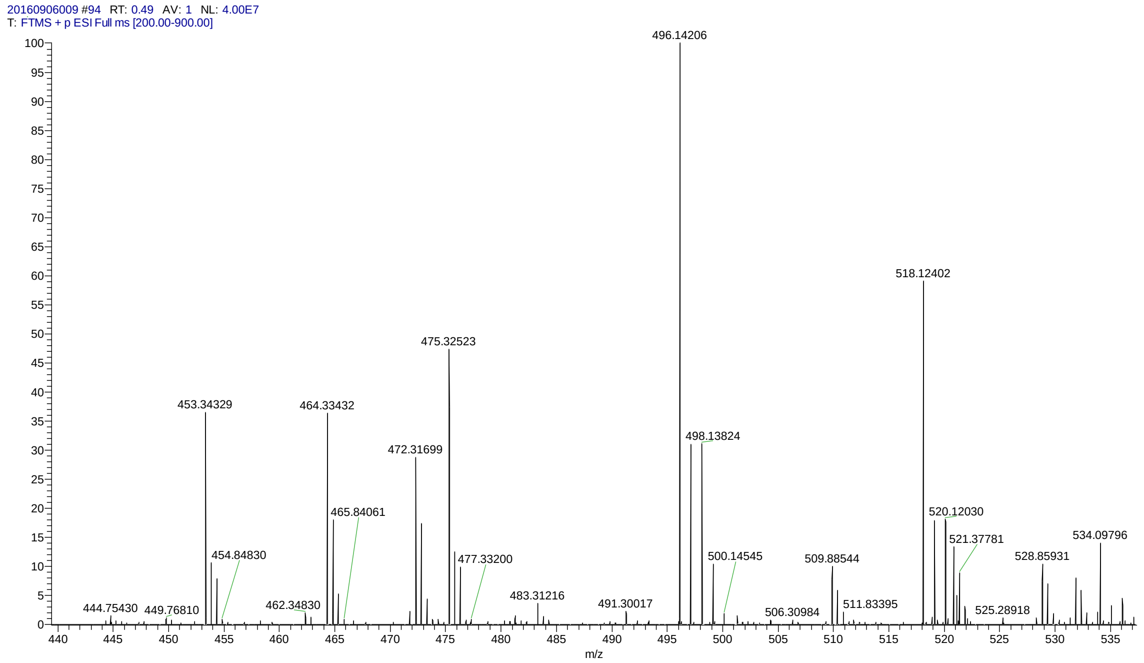
20160906009 #94 RT: 0.49 AV: 1 NL: 4.00E7
T: FTMS + p ESI Full ms [200.00-900.00]
496.14206
518.12402
475.32523
453.34329
464.33432
498.13824
472.31699
465.84061
520.12030
521.37781
534.09796
454.84830
477.33200
500.14545
509.88544
528.85931
483.31216
444.75430
449.76810
462.34830
491.30017
506.30984
511.83395
525.28918
m/z

5k

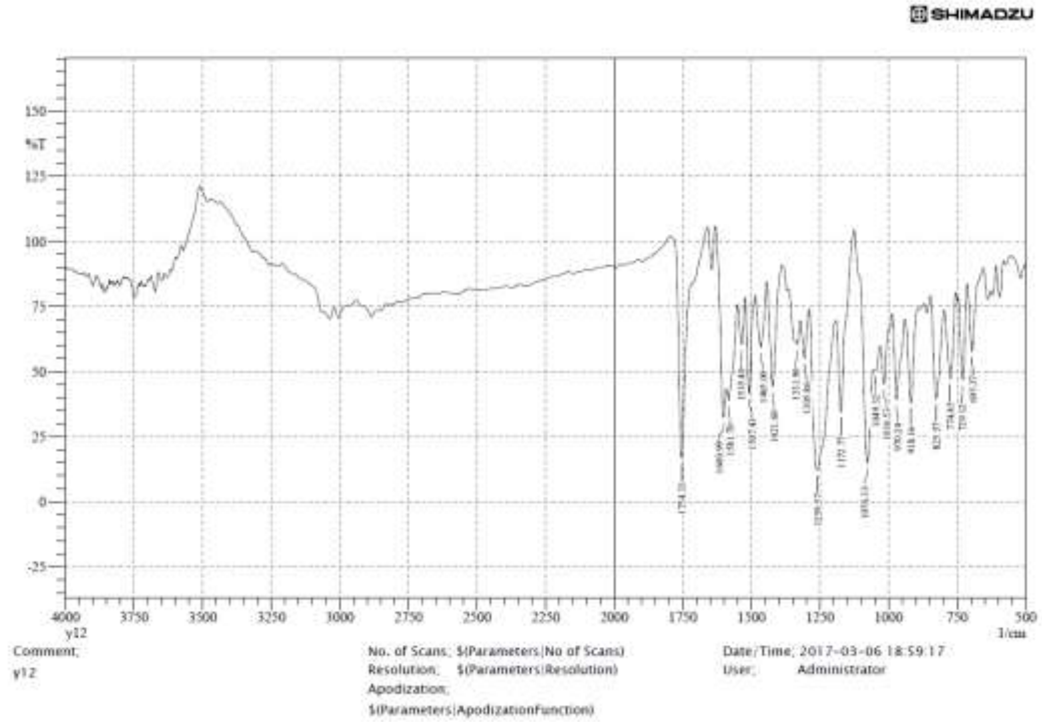
SHIMADZU
%T
1/cm
y12
Comment;
y12
No. of Scans; $(Parameters|No of Scans)
Resolution; $(Parameters|Resolution)
Apodization;
$(Parameters|ApodizationFunction)
Date/Time; 2017-03-06 18:59:17
User; Administrator

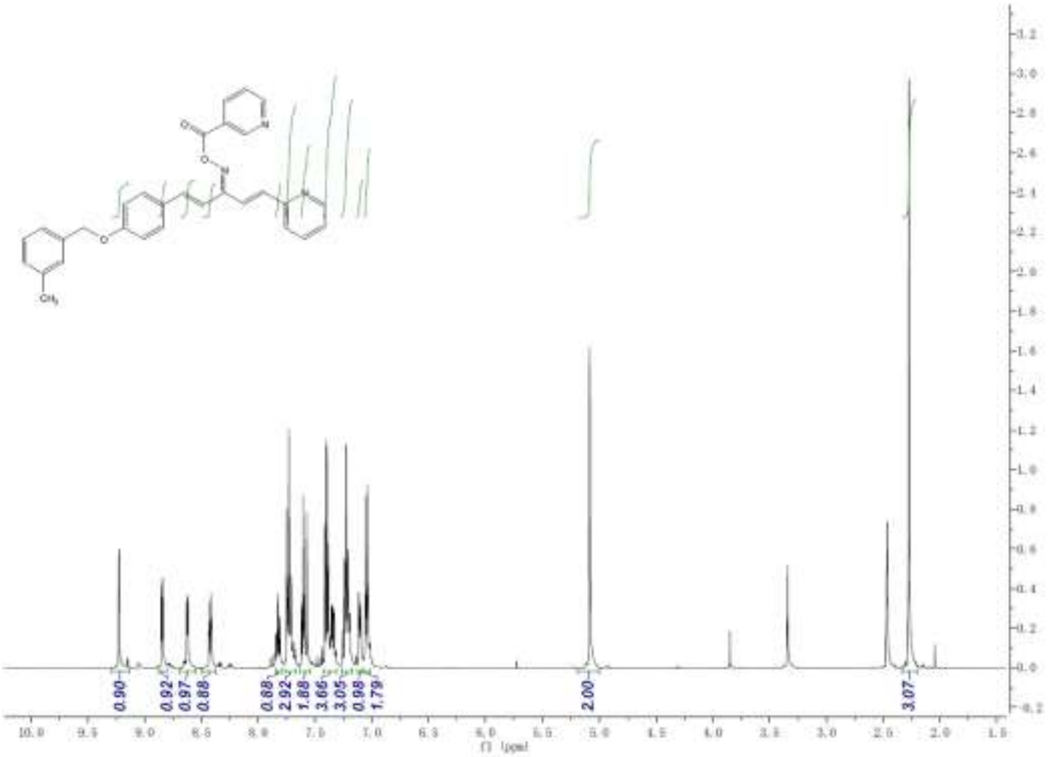
0.90
0.92
0.97
0.88
0.88
2.92
1.88
3.65
3.05
0.98
1.79
2.00
3.07
f1 (ppm)

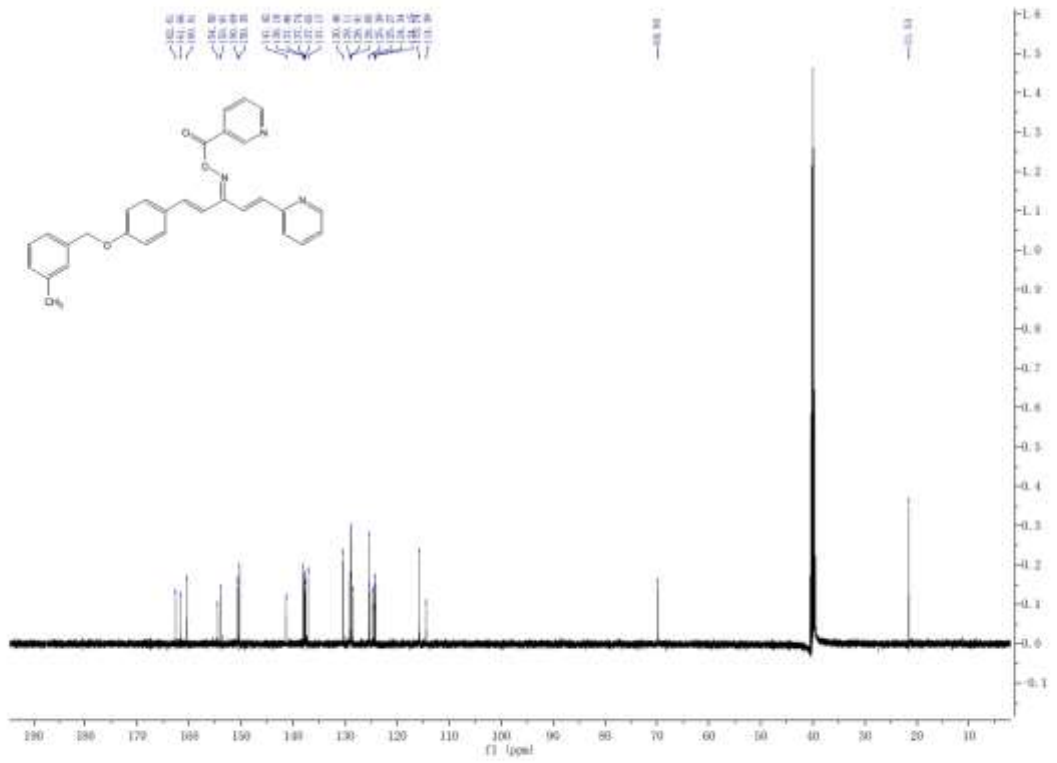

f1 (ppm)

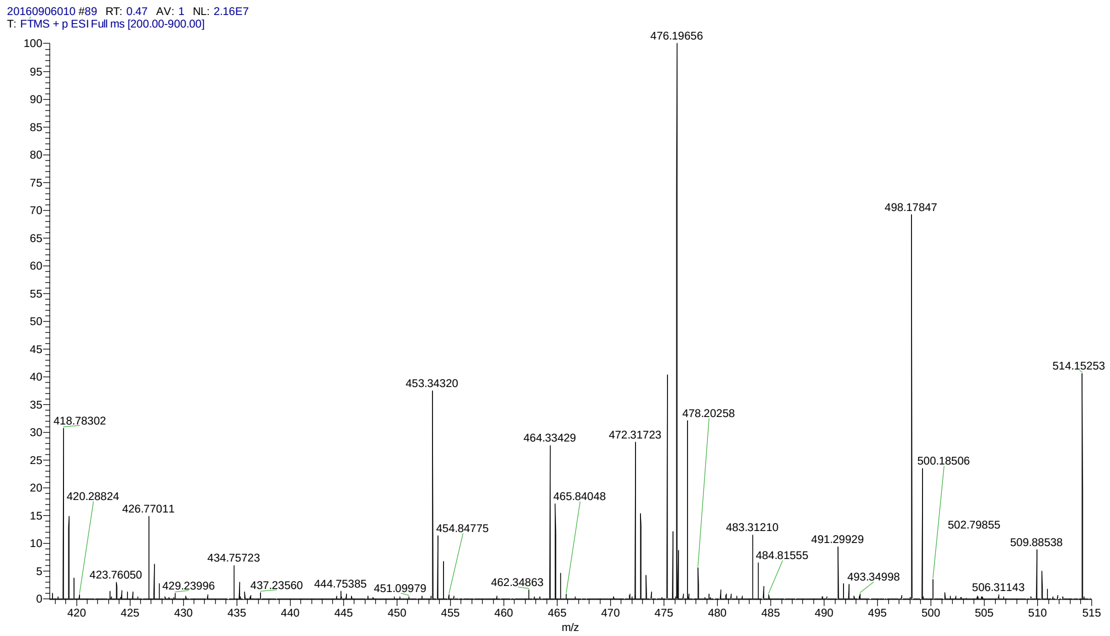

20160906010 #89 RT: 0.47 AV: 1 NL: 2.16E7
T: FTMS + p ESI Full ms [200.00-900.00]
476.19656
498.17847
514.15253
453.34320
418.78302
478.20258
464.33429
472.31723
500.18506
420.28824
465.84048
426.77011
454.84775
483.31210
502.79855
491.29929
509.88538
434.75723
484.81555
423.76050
493.34998
429.23996
437.23560
444.75385
451.09979
462.34863
506.31143
m/z
